# Instruments Used to Measure Sexual Identity Minority Stress: A COSMIN Systematic Review

**DOI:** 10.1007/s10508-026-03508-z

**Published:** 2026-08-28

**Authors:** Maria Misevic-Kallenbach, Susann Conrad, Simone Freitag, Anja Jacobs, Jacqueline Schirm, Madlen Sixtensson, Petra Warschburger

**Affiliations:** 1 Department of Psychology, Counseling Psychology, University of Potsdam, Karl-Liebknecht-Str. 24-25, 14476 Potsdam, Germany; 2 Independent Researcher, Berlin, Germany; 3 Institute of Nursing Care Science and Interprofessional Learning, University Medicine Greifswald, Greifswald, Germany; 4 The German Cancer Society, Berlin, Germany; 5 Independent Researcher, Schöneiche, Germany; 6 Independent Researcher, Potsdam, Germany

**Keywords:** Minority stress, Sexual identity, Lesbian, Gay, Sexual orientation

## Abstract

This systematic review evaluates the psychometric properties of instruments used to measure minority stress in lesbian, gay, bisexual, and queer (LGBQ) populations. A systematic search identified measurement instruments for dimensions of minority stress in adult LGBQ populations. The instruments were evaluated using the *CO*nsensus-based *S*tandards for the selection of health *M*easurement *IN*struments (COSMIN) methodology. Eight out of 105 instruments qualified for appraisal. The Lesbian, Gay, and Bisexual Identity Scale (LGBIS) and its German adaptation, LGBIS-DE, demonstrated the strongest psychometric properties and are recommended for use. However, the review indicated notable variation in how minority stress was conceptualized and operationalized across instruments. Additionally, content validity, as defined by COSMIN, has not been sufficiently explored, compromising the robustness of many instruments. This review highlights the need for greater standardization in the definition of minority stress constructs and a greater focus on content validity in future research. While the LGBIS and LGBIS-DE demonstrated strong psychometric properties, data were not available for all psychometric categories, particularly for reliability and responsiveness. Further research is needed to further validate the instruments included. PROSPERO Registration Number: CRD42021257995.

**Supplementary Information:**

The online version contains supplementary material available at https://doi.org/10.1007/s10508-026-03508-z.

Lesbian, gay, bisexual, and queer (LGBQ) individuals experience unique stressors related to societal stigma, discrimination, and internalized homonegativity, collectively referred to as minority stress. Meyer’s minority stress model (Meyer, 2003, 2015) posits that these stressors contribute to adverse mental health outcomes among sexual minority populations, including higher rates of suicide, anxiety, depression, and substance use disorders compared to heterosexual individuals (Blondeel et al., 2016; King et al., 2008; Pompili et al., 2014; Salway et al., 2019; Sandfort et al., 2006; Williams et al., 2021; Wittgens et al., 2022). The model differentiates between distal stressors, such as experiences of prejudice and discrimination, and proximal stressors, including internalized homonegativity and expectations of rejection, both of which contribute to the chronic strain experienced by LGBQ individuals. Research demonstrates that minority stressors also negatively impact physical health, with prejudice-related events posing significant risks beyond those associated with general life stressors (Frost et al., 2015). Unique resources, namely pride and community connectedness, mediate these processes among sexual minority individuals (Frost & Meyer, 2023; Meyer, 2015).

Recently, multidimensional and strengths-based perspectives have been emphasized, reorienting theoretical attention toward strengths and community-based resilience rather than individual deficits (Levitt et al., 2023; Perrin et al., 2020). Building on this shift, the temporal intersectional minority stress model situates minority stress within historical, cultural, and intersectional contexts and attends to developmental and life course timing as well as sociopolitical conditions (Rivas‐Koehl et al., 2023). In parallel, the psychological mediation framework specifies cognitive, affective, and interpersonal processes through which stigma-related stress confers risk for psychopathology, highlighting relevant pathways such as emotion dysregulation, rumination, rejection sensitivity, social isolation, and hopelessness (Hatzenbuehler, 2009). Extensions that incorporate rejection sensitivity further clarify how proximal stress translates into symptoms, including biases in attention and anticipatory anxiety (Pachankis et al., 2008), and delineate a temporal sequence from expectations of rejection to perceptions, cognitive and emotional responses, behavior, and outcomes (Feinstein, 2020). Empirical work underscores contextual variability across populations and settings, supporting models that are sensitive to these differences (McConnell et al., 2018; Moorhead et al., 2024).

Given the pivotal role of minority stress in explaining health disparities among sexual minority individuals (Institute of Medicine, 2011), various measurement instruments are used to assess different aspects of this concept. These instruments are critical for both research and clinical practice, facilitating the understanding of stressors, their mental health impacts, and the evaluation of interventions aimed at reducing stress.

The inconsistent conceptualization and operationalization of minority stress constructs across different instruments, often manifesting as jingle-jangle fallacies (Misevic-Kallenbach et al., 2026), complicate the selection of an appropriate measure for researchers and practitioners. A jingle fallacy occurs when instruments with similar names are mistakenly assumed to measure the same construct, while a jangle fallacy arises when instruments with different names are assumed to measure distinct constructs, despite their overlap (Pedhazur & Schmelkin, 1991). For example, several instruments have been used to assess internalized homophobia, yet they operationalize the construct differently: The Nungesser Homosexual Attitudes Inventory (Nungesser, 1983) incorporates items related to outness and disclosure, whereas later measures like the Revised Internalized Homophobia Scale (Herek et al., 1998) strictly limit the construct to self-directed negative affect, treating outness as a distinct behavioral variable. Furthermore, measurement validity is threatened when instruments conflate constructs that Meyer’s (Meyer, 2003, 2015) minority stress model treats as distinct. For instance, the Multifactor Internalized Homophobia Inventory (MIHI) (Flebus et al., 2012) nests “Community Connectedness” and “Outness” as sub-dimensions of internalized homophobia. Under Meyer’s framework, however, connectedness and outness are conceptualized as coping or resilience resources that mitigate stress, rather than being components of the stressor itself. Such conflation might render it difficult for researchers to disentangle the harmful effects of minority stress from the protective effects of community resilience.

These conceptual ambiguities are further compounded by inconsistencies in psychometric validation, particularly regarding content validity, structural validity, and reliability. As a result, the comparability of findings across studies is often limited, undermining the robustness of conclusions drawn from minority stress research. A more systematic evaluation of existing measurement instruments is therefore needed to identify the most psychometrically sound instruments, highlight gaps in the current measurement landscape, and provide clear recommendations for future research and instrument development.

This systematic review aims to address these challenges by rigorously assessing the psychometric properties of instruments used to measure aspects of minority stress in LGBQ populations. These instruments will be assessed and compared for their psychometric properties to identify appropriate measurement instruments in German and English. By evaluating the strengths and limitations of existing instruments, this review also aims to provide recommendations for future research and instrument development, thereby improving the assessment and understanding of minority stress in sexual identity minority individuals.

## Method

This systematic review was conducted and is reported in accordance with the PRISMA guideline for reporting systematic review of outcome measurement instruments (Elsman et al., 2024). A corresponding a priori protocol was registered on PROSPERO (ID: CRD42021257995).

### Data Sources and Study Selection

A literature search string (see Supplement 1) was developed by an experienced information scientist (JS) and subsequently peer-reviewed by a second information specialist. The systematic search was conducted across eight databases and last updated in February 2025. Additionally, a hand search in the GESIS databank was performed and the reference lists of included articles were examined for further relevant publications.

In a three-stage screening process, two independent reviewers (MMK, SC, SF, AJ, MS) identified psychometric studies on instruments measuring at least one dimension of minority stress in adult LGBQ populations, based on Meyer’s minority stress model. The initial selection was conducted by title and abstract, followed by full-text screening according to the following inclusion criteria:

- Population: Adult homosexual, bisexual, and queer persons
- Constructs measured with the instruments: Discrimination, Rejection, Victimization, Stigmatization, Internalized Homophobia, Negative Future Expectations, Concealment, Social Support, and Pride
- Type of instrument: Self-report questionnaires
- Types of studies: Psychometric studies.

A scoping review presents all instruments fulfilling these inclusion criteria (Misevic-Kallenbach et al., 2026). For the present systematic review, additional exclusion criteria were applied to isolate instruments measuring multiple dimensions of minority stress in English or German. Measurement instruments also had to be evaluated in samples including at least both gay and lesbian adults. This criterion aims to prioritize instruments that had been examined beyond a single sexual minority subgroup and a single gender group and were therefore, in principle, applicable across multiple sexual minority subgroups and gender groups. Accordingly, measurement instruments developed specifically for only one subgroup, such as bisexual individuals or a single gender group, were excluded. Instruments were therefore excluded if:

- E1: The instrument was not available in English or German.
- E2: The instrument addressed only a single dimension of minority stress (unidimensionality).
- E3: The target population did not include at least both lesbian and gay adults.
- E4: The target population focused only on a specific subgroup (e.g., military personnel).

The developers of all included instruments were contacted to request any additional data on psychometric evaluations, provided their contact information was available.

### Data Extraction

We utilized a data extraction form to capture study and measurement instrument characteristics, which was adapted from examples provided in the *CO*nsensus-based *S*tandards for the selection of health *M*easurement *IN*struments (COSMIN) manual (Mokkink et al., 2018). This form was piloted on one study included in the review. One author (MMK) extracted data on study characteristics, and another author (SC, SF, AJ, MS) checked the data for completeness and accuracy.

### Risk of Bias Assessment and Rating of Results

The risk of bias for the psychometric studies was assessed according to the COSMIN methodology (Mokkink et al., 2018; Terwee et al., 2018). Content validity, structural validity, internal consistency, cross-cultural validity, reliability, construct validity, and responsiveness were evaluated separately for risk of bias using the COSMIN Risk of Bias checklist, with two independent reviewers (MMK, SC, SF, AJ, MS) assessing each study. Conflicts were resolved by consensus, with discussions involving all reviewers when necessary. Each study was rated as “very good,” “adequate,” “doubtful,” or “inadequate,” with the lowest rating in any criterion determining the overall study rating. Psychometric properties were evaluated in line with the original authors’ hypotheses; for example, group comparison analyses were interpreted as evidence for cross-cultural or known-groups validity as specified by the authors.

The overall quality of the available evidence was assessed using the COSMIN-adapted Grading of Recommendations Assessment, Development and Evaluation (GRADE) (Mokkink et al., 2018; Terwee et al., 2018) framework and, when necessary, downgraded from high to moderate, low, or very low considering the risk of bias, inconsistency, imprecision, and indirectness (see Table S1 in Supplement 1).

Additionally, the results for each psychometric property were categorized as “sufficient” ( +), “insufficient” ( −), “inconsistent” ( ±), or “indeterminate” (?). The COSMIN criteria for good measurement criteria for each measurement property (Mokkink et al., 2018; Terwee et al., 2018) are presented in Table S2 in Supplement 1.

The final evaluation for each instrument included both the quality of evidence and the empirical support for each psychometric property.

Finally, the measurement instruments were classified into three distinct categories:

- Category A: Instruments with sufficient content validity and at least low-quality evidence for internal consistency. These instruments are recommended for use, as their results can be trusted.
- Category B: Instruments that do not meet the criteria for Category A or C. These instruments have potential but require further validation before they can be fully recommended. Additional research is needed to improve their evidence base.
- Category C: Instruments with high-quality evidence showing insufficient psychometric properties. These instruments are not recommended for use.

## Results

**Fig. 1:**
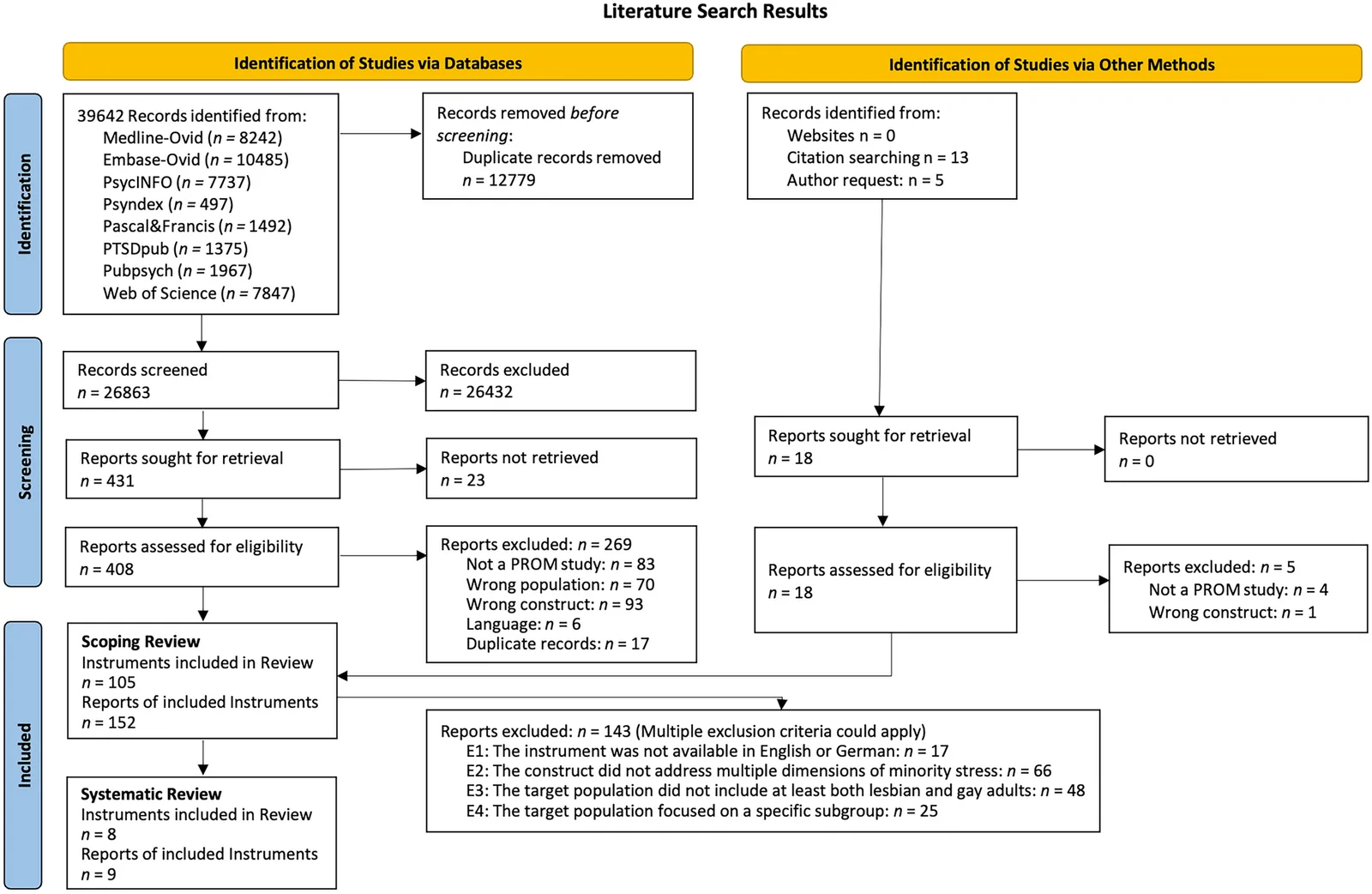
PRISMA 2020 flow diagram

The entire information retrieval process, from conducting systematic searches to the final inclusion of studies, is outlined in the accompanying flowchart presented in Fig. 1, following the PRISMA 2020 guidelines (Page et al., 2021). The systematic literature search yielded 39,642 records from all databases. After title-abstract screening and full-text screening, 152 references remained and screened for construct coverage across multiple dimensions of minority stress among both gay men and lesbian women and instrument language in English or German. In this last study selection step, 49% of the excluded reports met at least two exclusion criteria (see Fig. S1 in Supplement 1). The list of excluded studies is available in Supplement 2.

Seven measurement instruments were eligible for evaluation. The Gay and Lesbian Oppressive Situations Inventory (GALOSI) (Highlen et al., 2000) addresses frequency and effects through two distinct questionnaires, which were treated as separate instruments. For one instrument, a validated German version was identified and included for separate inspection. Thus, the total number of instruments evaluated was 8, namely:

1. The Daily Heterosexist Experiences Questionnaire (DHEQ) (Balsam et al., 2013)
2. The Multifactor Internalized Homophobia Inventory (MIHI) (Flebus et al., 2012)
3. The GALOSI-Frequency (GALOSI-F) (Highlen et al., 2000) and
4. The GALOSI-Effects (GALOSI-E) (Highlen et al., 2000)
5. The LGBT Minority Stress Measure (Outland, 2016)
6. The Lesbian and Gay Identity Scale (LGIS) (Mohr & Fassinger, 2000)
7. The Lesbian, Gay, and Bisexual Identity Scale (LGBIS) (Cramer et al., 2018; Mohr & Kendra, 2011) as well as
8. The German adaptation of the LGBIS (LGBIS-DE) (Gertzen et al., 2024; Niepel et al., 2019)

Regarding our author inquiries for additional information about the included measurement instruments, we received responses from five out of six contacted authors. We identified new psychometric data relevant to our research question in one publication (Gertzen et al., 2024).

Regarding the developmental path of the included instruments, the LGBIS (Cramer et al., 2018; Mohr & Kendra, 2011) is an updated version of the LGIS (Mohr & Fassinger, 2000). The original language of all instruments except the MIHI (Flebus et al., 2012) is English, with some translated versions. Recall periods varied, with some reflecting past experiences, others current states (see Table 1).

**Table 1:** Characteristics of the included measurement instruments

Instrument (Reference to First Article)	Construct(s)	Target population	Recall period	Subscales (Number of Items)	Response options	Range of scores/scoring	Original language	Available translations (Reference)
DHEQ (Balsam et al., 2013)	Minority stress	LGBQT	past 12 months ^a^	Gender expression (6), Vigilance (6), Parenting (6), Harassment and Discrimination (6), Vicarious trauma (6), Family of Origin (6), HIV/AIDS (5), Victimization (5), Isolation (4)	0 = did not happen/not applicable to me 1 = it happened, and it bothered me NOT AT ALL 2 = it happened, and it bothered me A LITTLE BIT 3 = it happened, and it bothered me MODERATELY 4 = it happened, and it bothered me QUITE A BIT 5 = it happened, and it bothered me EXTREMELY	NI	English	Polish (Mijas & Koziara, 2020)
MIHI (Flebus et al., 2012)	Internalized homophobia	Lesbian and Gay	NI	Fear of coming out (21), Regret about being homosexual (11), Moral condemnation (15), Gay–lesbian parenting (4), Integration in the homosexual community (7), Counter-prejudicial attitudes (7), Homosexual marriage (2), Stereotypes (7) ^b^	5-point Likert scales	NI	Italian	English
GALOSI-F (Highlen et al., 2000)	Frequencies of heterosexist situations	Lesbian and gay	NI	Couples Issues (4), Danger to Safety (5), Exclusion, Rejection and Separation (9), Internalized Homonegativity (10), Restricted Opportunities and Rights (3), Stigmatizing and Stereotyping (11), Verbal Harassment and Intimidation (7)	5-point Likert scales: 1 = Never 5 = Almost Always Not Applicable	NI	English	-
GALOSI-E (Highlen et al., 2000)	Effects of heterosexist situations	Lesbian and gay	NI	Couples Issues (3), Danger to Safety (6), Exclusion, Rejection and Separation (10), Internalized Homonegativity (9), Restricted Opportunities and Rights (3), Stigmatizing and Stereotyping (8), Verbal Harassment and Intimidation (8)	5-point Likert scales: 1 = None 2 = Slight 3 = Moderate 4 = Strong 5 = Extremely Strong Not Applicable	NI	English	-
LGBT Minority Stress Measure (Outland, 2016)	Minority stress	LGBQ	NI	Identity concealment (6), everyday discrimination/ microaggressions (13), rejection anticipation (6), discrimination events (6), Internalized stigma (7), victimization events (7), community connectedness (5)	1 = never 2 = rarely 3 = occasionally 4 = often 5 = all of the time OR 1 = strongly disagree 2 = disagree 3 = neither disagree nor agree 4 = agree 5 = strongly agree	1—5/ Higher scores indicate greater minority stress	English	Nigerian English (Ogunbajo et al., 2020)
LGIS (Mohr & Fassinger, 2000)	Beliefs and feelings related to LG identity	Lesbian and Gay	NI	Need for Privacy (6), Need for Acceptance (5), Internalized Homonegativity (5), Difficult Process (5), Identity Confusion (4), Superiority (2)	7-point scale: 1 = disagree strongly 7 = agree strongly	NI	English	-
LGBIS (Mohr & Kendra, 2011)	Concealment and negative Identity	LGBQT	Current state (“now”)	Acceptance Concerns (3), Concealment Motivation (3), Identity Uncertainty (4), Internalized Homonegativity (3), Difficult Process (3), Identity Superiority (3), Identity Affirmation (3), Identity Centrality (5)	1 = disagree strongly 2 = disagree 3 = disagree somewhat 4 = agree somewhat 5 = agree 6 = agree strongly	NI	English	German (Niepel et al., 2019), Turkish (Kemer et al., 2017), Portuguese (Oliveira et al., 2012), Czech (Pitonak & Cihak, 2023)

DHEQ: The Daily Heterosexist Experiences Questionnaire; GALOSI-E: Gay And Lesbian Oppressive Situations Inventory-Effects; GALOSI-F: Gay And Lesbian Oppressive Situations Inventory-Frequency; LGBIS: Lesbian, Gay, and Bisexual Identity Scale; LGBIS-DE: Lesbian, Gay, and Bisexual Identity Scale-German; LGB(QT): Lesbian, gay, bisexual (or queer or trans); LGIS: Lesbian and Gay Identity Scale; MIHI: The Multifactor Internalized Homophobia Inventory; and NI: no information

^a^ The authors provide alternative response categories (i.e., did happen/did not happen) and recall periods (i.e., daily, weekly)

^b^ Flebus et al. (2012) performed a factor analysis resulting in a 66-item and a 77-item solution. Due to the insignificant differences between these versions, only the latter is described further. It is inconclusive whether the 77-item questionnaire version represents the final version of the MIHI

**Fig. 2:**
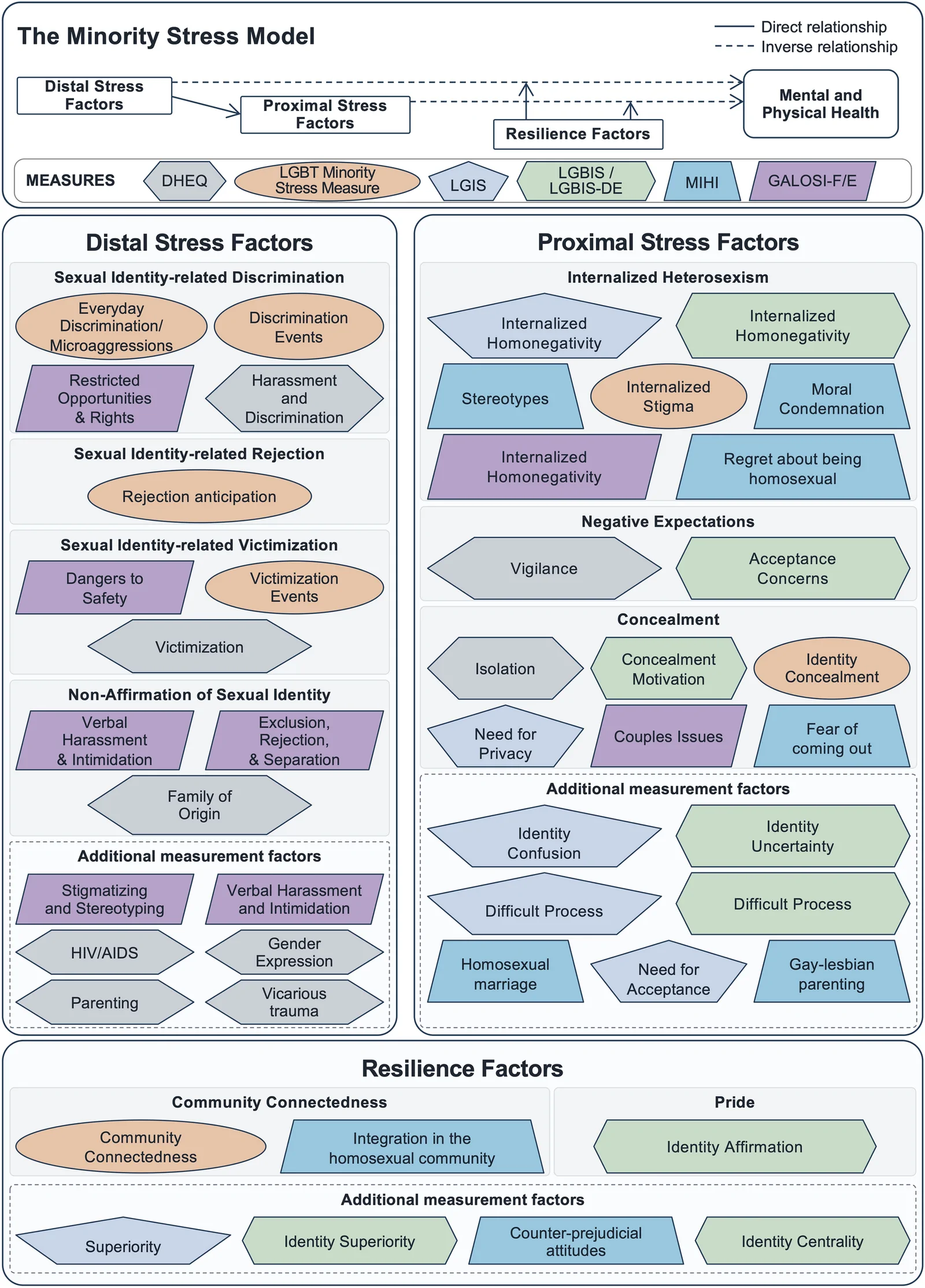
Instrument constructs embedded in Meyer’s Minority stress model

The main measurement constructs included minority stress, beliefs and feelings related to lesbian/gay identity, concealment and negative identity, and internalized homophobia as well as frequencies and effects of heterosexist situations. Figure 2 illustrates how the subscales of the evaluated measurement instruments are embedded within Meyer’s minority stress model. The figure maps the constructs measured by each instrument’s subscales onto the key dimensions of the model, specifically distal stress factors (e.g., discrimination, victimization), proximal stress factors (e.g., internalized homonegativity, concealment), and resilience factors (e.g., community connectedness, pride). A notable observation is the uneven distribution of constructs across the instruments, highlighting varying degrees of alignment with the minority stress model:

- The LGBT Minority Stress Measure (Outland, 2016) demonstrates the broadest coverage, with subscales capturing distal and proximal stress factors, as well as resilience factors.
- The DHEQ (Balsam et al., 2013) and GALOSI-F/E (Highlen et al., 2000) primarily focus on distal stress factors but also include subscales referring to proximal stress factors.
- The LGIS (Mohr & Fassinger, 2000) and LGBIS/LGBIS-DE (Cramer et al., 2018; Gertzen et al., 2024; Mohr & Kendra, 2011; Niepel et al., 2019) predominantly assess proximal stress factors while incorporating resilience factors.
- The MIHI (Flebus et al., 2012), despite its primary focus on internalized homophobia as a proximal stressor, extends its scope to include additional proximal stress factors and resilience factors.

Regarding psychometric validation studies, sample sizes ranged from 521 to 1,217 participants, aged 18 to 76, and included a mix of cisgender, transgender, and genderqueer individuals, identifying as lesbian, gay, bisexual, and queer. Recruitment occurred mainly online and through LGBTQ+ organizations, with studies conducted in the USA, Germany, and Italy. Response rates ranged from 49 to 98% (see Table 2).

**Table 2:** Characteristics of the included study populations

		Population^a^				Instrument administration			
Instrument	Reference	N	Age (yr) M (SD, range)	Gender	Sexual identity	Recruiting setting	Country	Language	Response rate
DHEQ	Balsam et al. (2013)	Study 2: 900 Study 3: 1217	Study 2: 34.0 (11.2, 18–76) Study 3: 36.6 (11.8, 18–74)	Study 2: 31% cis-male, 57% cis-female, 4% trans-female, 3% trans-male, 4% other Study 3: 32% cis-male, 51% cis-female, 6% trans-female, 3% trans-male, 4% genderqueer, 4% other	Study 2: 49% lesbian or gay, 32% bisexual, 12% queer, 12% two- spirit, 6% other Study 3: 26% gay, 31% lesbian, 22% bisexual, 10% queer, 2% two-spirit, 9% other	Online	USA	English	Study 2: NI Study 3: 70%
MIHI	Flebus et al. (2012)	1077	NI	NI	78% homosexual, 11% bisexual, 8% “almost exclusively heterosexual,” 1% no preference, < 1% no answer	Homosexual meeting places, personal contacts, university students	Italy	Italian	NI
GALOSI-F	Highlen et al. (2000)	Study 1: 607 Study 2: 300	Study 1: 32.1 (14.3, 18–75) Study 2: 33.2 (14.3, 18–76)	Study 1: 56% male, 39% female Study 2: 56% male, 38% female	Study 1: 56% gay, 39% lesbian Study 2: 56% gay, 38% lesbian	Announcements in university facilities, Snowball-sampling locally and by electronic mail, Internet chat rooms	USA	English	Study 1: NI ^b^ Study 2: 2: 98%
GALOSI-E									
LGBT Minority Stress Measure	Outland (2016)	640	NI (NI, 18–55 +)	44% cis-male, 36% cis-female, 7% trans-female, 5% trans-male, 9% other	34% gay, 17% lesbian, 31% bisexual, 18% other	Online	USA	English	91%
LGIS	Mohr and Fassinger (2000)	1004	36.6 (9.5, 18–69)	41% male	59% lesbian, 41% gay	LG organizations and businesses in East Coast cities, print advertisements and events in Washington D.C	USA	English	49%
LGBIS	Mohr and Kendra (2011)	Study 1: 795 Study 2: 51	Study 1: 22.9 (5.6, 18–52) Study 2: 22.7 (5.1, 18–45)	Study 1: 51% cis-female, 46% cis-male, 1% trans-male, < 1% other, < 1% trans-female Study 2: 67% female, 33% male	Study 1: 30% lesbian, 40% gay, 30% bisexual Study 2: 57% lesbian, 25% gay, 14% bisexual, 4% queer	LGB student organizations at universities in North America	USA	English	82%
	Cramer et al. (2018)	521	40.2 (13.8, NI)	37% cis-male, 50% cis-female, 3% trans-female, 3% trans-male, 6% gender queer	21% gay, 4% lesbian, 47% bisexual, 29% no label/ no preference/ fluid/ queer	BDSM practitioners	USA	English	91%
LGBIS-DE	Niepel et al. (2019)	Germany: 1190 USA: 435	Germany: 27.8 (6.2, 18–40) USA: 23.4 (6,0, 18–52)	Germany: 53% male, 47% female USA: 56% male, 44% female	Germany: 53% gay, 47% lesbian USA: 56% gay, 44% lesbian	Germany: online, gay/ lesbian institutions USA	Germany, USA	German, English	NI
	(Gertzen et al., 2024)	759	NI ^c^	98% cis-male 2% trans-male	NI ^d^	Online advertisements and newsletters	Germany	German	NI

BDSM: bondage and discipline, dominance and submission, and sadomasochism; DHEQ: The Daily Heterosexist Experiences Questionnaire; GALOSI-E: Gay And Lesbian Oppressive Situations Inventory-Effects; GALOSI-F: Gay And Lesbian Oppressive Situations Inventory-Frequency; LGBIS: Lesbian, Gay, and Bisexual Identity Scale; LGBIS-DE: Lesbian, Gay, and Bisexual Identity Scale-German; LGIS: Lesbian and Gay Identity Scale; M: Mean; MIHI: The Multifactor Internalized Homophobia Inventory; NI: No Information; SD: standard deviation; and yr: years

^a^Refers to quantitative studies

^b^The response rates are reported as “poor” and hindered conducting an exploratory factor analysis

^c^53% of the individuals in the non-chemsex group (*n* = 652) and 79% of the chemsex group (*n* = 107) were aged between 30 and 49

^d^The study population consists of men who have sex with men

### Psychometric Properties

Table 3 presents the results regarding the psychometric properties of the included measurement instruments. The inter-rater reliability of the reviewers, assessed using weighted Cohen’s kappa, was 0.73 (95% CI: 0.54; 0.91), indicating substantial agreement according to Landis and Koch’s (1977) Benchmark Scale. Disagreements were solved by consensus through discussion.

**Table 3:** Psychometric properties and quality of evidence for each measurement instrument

	DHEQ (Balsam et al., 2013)		MIHI (Flebus et al., 2012)		GALOSI-F/E ^a^ (Highlen et al., 2000)		LGBT Minority Stress Measure (Outland, 2016)		LGIS (Mohr & Fassinger, 2000)		LGBIS (Cramer et al., 2018; Mohr & Kendra, 2011)		LGBIS-DE (Gertzen et al., 2024; Niepel et al., 2019)	
	Overall Rating ^b^	QoE^ c^	Overall Rating ^b^	QoE^ c^	Overall Rating ^b^	QoE^ c^	Overall Rating ^b^	QoE^ c^	Overall Rating ^b^	QoE^ c^	Overall Rating ^b^	QoE^ c^	Overall Rating ^b^	QoE^ c^
Content validity	?	Very low	?	Very low ^d^	+	Very low ^d^	?	Very low ^d^	+	Very low ^d^	+	Very low ^d^	+	Very low ^d^
*Relevance*	?	Low	?	Very low ^d^	?	Very low ^d^	?	Very low ^d^	+	Very low ^d^	+	Very low ^d^	+	Very low ^d^
*Comprehensiveness*	+	Low	+	Very low ^d^	+	Very low ^d^	?	Very low ^d^	?	Very low ^d^	?	Very low ^d^	+	Very low ^d^
*Comprehensibility*	?	Very low ^d^	?	Very low ^d^	+	Very low ^d^	+	Very low ^d^	+	Very low ^d^	+	Very low ^d^	+	Very low ^d^
Structural validity	-	Moderate	-	Moderate	- ^e^	Low ^e^	-	High	-	Moderate	+	High	+	Moderate
Internal consistency	?	High	?	Low	? ^e^	Low ^e^	?	High	?	Moderate	+	High	+	High
Cross-cultural validity													+	Low
Reliability											?	Low		
Construct validity	+	Moderate					+	Moderate	+	Low	+	High	+	Moderate

DHEQ: The Daily Heterosexist Experiences Questionnaire; GALOSI-E: Gay And Lesbian Oppressive Situations Inventory-Effects; GALOSI-F: Gay And Lesbian Oppressive Situations Inventory-Frequency; LGBIS: Lesbian, Gay, and Bisexual Identity Scale; LGBIS-DE: Lesbian, Gay, and Bisexual Identity Scale-German; LGIS: Lesbian and Gay Identity Scale; LGBT: Lesbian, gay, bisexual and trans; MIHI: The Multifactor Internalized Homophobia Inventory; and QoE: Quality of Evidence

^a^The GALOSI-F and GALOSI-E were treated as separate instruments and evaluated independently. However, as they produced identical results, they are presented together

^b^Sufficient ( +), insufficient (−), inconsistent (±), or indeterminate (?)

^c^High, moderate, low, or very low

^d^Due to insufficient or missing evidence, as determined by the authors’ ratings in accordance with the COSMIN methodology (Terwee et al., 2018). Rating criteria are presented in Table S2 in Supplement 1

^e^It is unclear whether the measurement model of the GALOSI-F/E is formative or reflective. Following the COSMIN methodology (Mokkink et al., 2018), we assumed a reflective structure and rated the presented exploratory factor analysis and Cronbach’s α

### Content Validity

Content validity refers to how well a measurement instrument comprehensively captures all relevant aspects, ensuring its content accurately reflects the construct being measured.

Across the eight instruments, most instruments exhibit very low-quality evidence for content validity. Only data of the DHEQ (Balsam et al., 2013) allowed an evaluation of relevance and comprehensiveness. As an initial development step, the authors conducted 12 qualitative focus groups and 17 semi-structured interviews (Balsam et al., 2013). However, the available data did not fully meet the COSMIN requirements: Specifically, it was unclear whether the interviewers or moderators had received prior training and whether saturation was achieved during data collection. As a result, the quality of the instrument design was assessed as “doubtful.”

In terms of relevance, the study (Balsam et al., 2013) did ensure that the included items were relevant to both the construct of interest (COSMIN criterion 1) and the target population (COSMIN criterion 2). However, the context of use for the instrument remained unclear, meaning it was not possible to determine whether the items were appropriate for that specific context (COSMIN criterion 3). In addition, the relevance of the response options (COSMIN criterion 4) and the recall period (COSMIN criterion 5) was not examined. As evidence and results were therefore only available for two of the five COSMIN assessment criteria, the overall relevance was determined as indeterminate (?) based on low-quality evidence.

Comprehensiveness of the DHEQ was assessed in a quantitative study using an open-ended item. However, it is unclear whether the comprehensiveness of the final item selection was examined, leading to a “doubtful” rating for the quality of this study. The study’s results were rated as sufficient ( +), resulting in an overall sufficient rating for comprehensiveness based on low-quality evidence. This suggests that, although not fully investigated, all key concepts are likely included in the DHEQ.

For all other instruments, following the COSMIN methodology (see Table S2 in Supplement 1), a rating of reviewers was used to make a judgment on the overall rating (Terwee et al., 2018). Reflecting lacking or inadequate evidence, the quality of evidence for all instruments was set to very low. Most instruments received at least one indeterminate rating (?) on content validity, indicating that it is unclear whether they accurately measure all relevant dimensions of minority stress comprehensively. An exception is the LGBIS-DE (Niepel et al., 2019) receiving exclusively sufficient ( +) ratings, although based on very low quality of evidence.

### Structural Validity

Structural validity evaluates whether the instrument’s dimensions align with the theoretical construct it intends to measure. As noted in the COSMIN manual (Mokkink et al., 2018), this assessment is relevant only for measurement instruments based on a reflective model, where all items in a scale or subscale represent a single underlying construct and are expected to be correlated.

The quality of evidence for the LGBT Minority Stress Measure (Outland, 2016) and the LGBIS (Cramer et al., 2018; Mohr & Kendra, 2011) was judged to be high. While the LGBIS (Cramer et al., 2018; Mohr & Kendra, 2011) also performed well in regard to structural validity receiving a sufficient rating ( +), the results for the LGBT Minority Stress Measure (Outland, 2016) were judged to be insufficient (−). The confirmatory factor analysis (CFA) of the LGBT Minority Stress Measure (Outland, 2016) yielded a comparative fit index (CFI) of 0.911, a standardized root-mean-square residual (SRMR) of 0.06, and a root-mean-square error of approximation (RMSEA) of 0.06. However, these values did not meet the COSMIN criteria for CFA, which require a CFI (or comparable measure) > 0.95, RMSEA < 0.06, and SRMR < 0.08 to indicate a good model fit (see Table S2 in Supplement 1). A CFI below 0.95 indicates that the model does not adequately explain the variance in the data, while the RMSEA and SRMR values, although close, are at the threshold of acceptability, indicating potential room for improvement in model fit.

For the LGIS (Mohr & Fassinger, 2000), structural validity was rated insufficient ( −) with moderate certainty of evidence. The CFA yielded CFI = 0.91, which does not meet COSMIN thresholds for good fit. Certainty of evidence was downgraded for indirectness according to GRADE. Our systematic review targets LGBQ adults, whereas the available validation samples comprised gay men and lesbian women; thus, applicability to bisexual populations is uncertain.

The DHEQ (Balsam et al., 2013) and MIHI (Flebus et al., 2012) provided moderate quality of evidence for insufficient structural validity (-), while the GALOSI-F and GALOSI-E (Highlen et al., 2000) were rated similarly based on low quality of evidence. For these instruments, only exploratory factor analyses (EFAs) were available, therefore not fulfilling the COSMIN requirement of CFA.

The LGBIS-DE (Niepel et al., 2019) demonstrated sufficient structural validity ( +) based on moderate quality of evidence. The evidence for the LGBIS-DE was rated down to moderate for indirectness, as the psychometric studies included only cisgender gay men and lesbian women. The scope of this systematic review as well as the target population of the LGBIS-DE also includes bisexual individuals and is not restricted to cisgender people.

### Internal Consistency

Internal consistency evaluates whether the items within each scale of the instrument are sufficiently correlated, providing an underlying reflective measurement model.

Following the COSMIN methodology (Mokkink et al., 2018) (see Table S2 in Supplement 1), the rating on internal consistency depended on the previous rating on structural validity because unidimensionality is required to be proven for a sensible interpretation of internal consistency analyses. Therefore, if not at least low evidence for sufficient structural validity was available, the internal consistency rating was set to indeterminate (?). This was the case for the DHEQ (Balsam et al., 2013), LGBT Minority Stress Measure (Outland, 2016), LGIS (Mohr & Fassinger, 2000), MIHI (Flebus et al., 2012), and GALOSI-F and E (Highlen et al., 2000) which all received insufficient ratings (−) on structural validity.

The LGBIS (Mohr & Kendra, 2011) and LGBIS-DE (Gertzen et al., 2024; Niepel et al., 2019) both received sufficient ratings based on high quality of evidence. For this criterion, the evidence for the LGBIS-DE was not rated down to moderate for indirectness because the study by Gertzen et al. (2024) provides additional evidence for men who have sex with men.

### Cross-Cultural Validity

Cross-cultural validity assesses whether a translated or adapted instrument measures the same construct as the original across diverse cultural groups, including differences in ethnicity, language, gender, age, or patient populations.

Data are available for the German LGBIS-DE, which examined measurement invariance across language/nationality and gender groups (Niepel et al., 2019). The scale demonstrated sufficient ( +) measurement invariance, although based on low-quality evidence. The evidence was downgraded for risk of bias and indirectness. Niepel et al., 2019 reported that the samples were similar in relevant characteristics except for the group variable, but did not provide supporting evidence, leading to an “adequate” rating on study quality according to COSMIN methodology (Mokkink et al., 2018).

### Reliability

Reliability refers to the extent to which the total variance in measurements reflects true differences between individuals.

There are very limited data on the reliability of the included measurement instruments. Only the LGBIS (Mohr & Kendra, 2011) shows low quality of evidence for indeterminate reliability (?). The instrument developers reported Pearson correlation coefficients instead of intraclass correlation coefficients (ICC) for test–retest reliability but did not provide evidence confirming the absence of systematic changes over time. Consequently, this led to a “doubtful” rating for the quality of evidence and an indeterminate (?) rating for the measurement properties according to the COSMIN methodology (Mokkink et al., 2018).

### Construct Validity

Construct validity evaluates the extent to which the instrument is related to other variables in a manner consistent with theoretical expectations.

All instruments with available data for this measurement property demonstrate sufficient ( +) construct validity. However, the quality of evidence varies, ranging from low for the LGIS (Mohr & Fassinger, 2000) to moderate for the DHEQ (Balsam et al., 2013), LGBT Minority Stress Measure (Outland, 2016), and LGBIS-DE (Niepel et al., 2019) and high for the LGBIS (Cramer et al., 2018; Mohr & Kendra, 2011).

In the case of the LGIS (Mohr & Fassinger, 2000), the quality for assessing construct validity was rated as “adequate,” leading to a downgrade in the quality of evidence. Sufficient measurement properties of the comparator instruments were presented but were mostly not derived from external studies in lesbian and gay populations. The evidence was further downgraded for indirectness, as the sample included only gay and lesbian individuals, whereas the scope of this review also includes bisexual individuals.

For the DHEQ (Balsam et al., 2013), the convergent validity analysis lacked sufficient justification for the measurement properties of the comparator instruments. Furthermore, the subgroups for known-groups validity differed in key demographic variables beyond the intended group characteristic. Consequently, the two studies received a “doubtful” rating, leading to a GRADE assessment of moderate evidence quality.

For the LGBT Minority Stress Measure (Outland, 2016), sufficient measurement properties of the comparator instruments were provided, though their relevance to the study population is unclear. Additionally, Pearson correlations were calculated, but the distribution of scores (SD) was not reported, resulting in an “adequate” study rating. For the LGBIS-DE (Niepel et al., 2019), the evidence was downgraded to moderate due to indirectness.

### Recommendations on Use of Measurement Instruments

As the final step in the COSMIN appraisal, the eight instruments are classified into categories A (“recommended”), B (“additional research needed”), and C (“not recommended”) on the basis of the summarized ratings across measurement properties (see Table 4).

**Table 4:** Classification of measurement instruments based on psychometric properties

Measurement Instrument (Reference)	Category A^a^: Any QoE for Content Validity + and ≥ low QoE for Internal Consistency +	Category B^a^: Neither A nor C	Category C^a^: High QoE for any -
DHEQ (Balsam et al., 2013)		x	
MIHI (Flebus et al., 2012)		x	
GALOSI-F (Highlen et al., 2000)		x	
GALOSI-E (Highlen et al., 2000)		x	
LGBT Minority Stress Measure (Outland, 2016)			x
LGIS (Mohr & Fassinger, 2000)		x	
LGBIS (Cramer et al., 2018; Mohr & Kendra, 2011)	x		
LGBIS-DE (Gertzen et al., 2024; Niepel et al., 2019)	x		

DHEQ: The Daily Heterosexist Experiences Questionnaire; GALOSI-E: Gay And Lesbian Oppressive Situations Inventory-Effects; GALOSI-F: Gay And Lesbian Oppressive Situations Inventory-Frequency; LGBIS: Lesbian, Gay, and Bisexual Identity Scale; LGBIS-DE: Lesbian, Gay, and Bisexual Identity Scale-German; LGIS: Lesbian and Gay Identity Scale; LGBT: Lesbian, gay, bisexual and trans; MIHI: The Multifactor Internalized Homophobia Inventory; and QoE: Quality of Evidence

^a^Measurement instruments in category “A” are suitable for use, and their results can be trusted. Instruments in category “B” show potential but require further research to confirm their quality. Instruments in category “C” are not recommended for use (Mokkink et al., 2018; Terwee et al., 2018)

The LGBIS-DE (Niepel et al., 2019) and the original LGBIS (Mohr & Kendra, 2011) are the most suitable instruments for assessing aspects of minority stress in LGBQ populations, categorized as Category A. Both instruments demonstrate strong content validity, internal consistency, and structural validity, supported by evidence ranging from very low to high quality. These instruments are highly recommended for both research and clinical use. However, future studies should aim to address gaps in reliability and, for the LGBIS-DE, include a broader target population in psychometric validations. Further research could also explore the responsiveness of these scales over time.

The DHEQ (Balsam et al., 2013) falls into Category B. Evidence for content validity is indeterminate, and its structural validity as well as internal consistency requires further validation. Instruments such as the MIHI (Flebus et al., 2012) and the GALOSI-F and GALOSI-E (Highlen et al., 2000) are also in Category B and require further research to fully validate their use, particularly regarding their structural validity for the MIHI (Flebus et al., 2012).

The LGIS (Mohr & Fassinger, 2000), classified in Category B, has been a valuable tool for assessing aspects of minority stress early on. However, the development of the LGBIS (Cramer et al., 2018; Mohr & Kendra, 2011) has led to improved psychometric properties across key areas. Therefore, the LGBIS, placed in Category A, is a more robust choice.

The LGBT Minority Stress Measure (Outland, 2016) is placed in Category C due to insufficient psychometric properties, particularly for its structural validity. It is not recommended for use until substantial improvements are made.

## Discussion

This systematic review provides a comprehensive evaluation of eight measurement instruments assessing aspects of minority stress in LGBQ populations. The systematic assessment of these tools offers a valuable foundation for improving the quality and consistency of measurement in future studies. This review fills a critical gap in the existing literature, as there is currently no consensus on how to operationalize and measure minority stress. The heterogeneity of theoretical foundations and inconsistent use of terminology have limited the comparability of findings across studies and complicated the interpretation of results.

### Jingle-Jangle Fallacies in Minority Stress Measurement

Only two of the eight instruments explicitly identified minority stress as their main construct, revealing substantial variability in construct conceptualization. In this review, we assumed that measurement instruments with at least two subscales directly related to the dimensions of Meyer’s (2003, 2015) minority stress model can be used to measure aspects of minority stress, irrespective of the measurement instruments’ original intent. The remaining six instruments tend to focus on selected dimensions of Meyer’s model, mostly in line with their intended construct. For example, GALOSI-F and GALOSI-E (Highlen et al., 2000) primarily assess distal stressors while additionally capturing proximal stressors, and the MIHI (Flebus et al., 2012) focuses on internalized heterosexism while also assessing concealment and resilience. These findings align with a prior scoping review (Misevic-Kallenbach et al., 2026), which highlighted inconsistencies in construct operationalizations. Our review builds upon this by demonstrating the need for greater conceptual clarity in minority stress measurement. As Morrison et al. (2016) also observed in their study of discrimination measures for sexual minority populations, a more standardized approach to construct definition is crucial for achieving better generalizability across studies.

Across instruments, we noted substantial overlap between subscales. Most subscales primarily target proximal stressors. Notably, the LGBT Minority Stress Measure (Outland, 2016) was the only instrument covering all domains of Meyer’s (2003, 2015) minority stress model, but it did not demonstrate sufficiently robust psychometric properties in our COSMIN appraisal. Conversely, the highest-rated instrument, the LGBIS (Mohr & Kendra, 2011), showed strong coverage of proximal stressors and included resilience-related domains, yet it does not assess distal stressors. At the same time, this pattern may be appropriate for some research aims if the intended construct is focused on proximal stress processes. Taken together, these findings underscore the importance of specifying the intended measurement construct(s) a priori and selecting the best-fitting tool accordingly. In light of the proliferation of existing measurement instruments and ongoing conceptual challenges (Misevic-Kallenbach et al., 2026), we suggest prioritizing targeted psychometric refinement and, where needed, construct-aligned extensions of existing instruments.

In light of the diversity of perspectives and study aims, and in acknowledgment of the fact that comprehensive standardization of measurement constructs may not be attainable, we encourage authors to specify the target construct a priori, state the theoretical foundations, and define the facets intended for operationalization. Decentralized construct taxonomies offer a structured approach by providing explicit construct operationalizations and methodological guidance for measurement (Peters & Crutzen, 2024). By comprehensive construct definitions, it becomes possible to delineate competing definitions and to identify the most empirically supported specification (Peters & Crutzen, 2024). At the same time, given the current lack of conceptual clarity in the field, it cannot yet be determined conclusively what should be classified as a jingle or a jangle fallacy. Given the importance and urgent need for health research in LGBQ populations, it is critical that this research is not hindered by limited generalizability caused by inconsistencies in measurement constructs.

### Quality of Evidence and Rating of Psychometric Properties

The results from the psychometric assessment highlight significant gaps in the psychometric properties of many of these instruments, especially in terms of content validity, structural validity, and internal consistency. These findings point to important limitations in the current tools used to measure aspects of minority stress. A major issue identified in this review is the lack of high-quality evidence for content validity. In the COSMIN methodology, this is particularly worrisome as content validity is considered to be most important measurement property (Terwee et al., 2018). In the context of minority stress, which involves complex and multifaceted experiences related to stigma, discrimination, and internalized homonegativity (Meyer, 2003, 2015), it is critical for measurement tools to reflect these dimensions comprehensively.

Most instruments lacked high-quality qualitative development, relying heavily on theoretical frameworks and literature reviews rather than empirical research. While valuable, these methods may not fully capture the lived experiences of minority stress processes. One possible explanation for this gap may be that relevant qualitative studies may have been conducted but not published. However, this was not confirmed in our correspondence with the developers of the LGIS, LGBIS, LGBIS-DE, and MIHI (Flebus, Niepel, & Mohr, personal communications, September–October 2024).

Without qualitative development studies, assessing the content validity of these tools remains a challenge. In this regard, standardized publication of qualitative research within the instrument development process would be invaluable. Introducing a structured checklist as part of the submission process for instrument development papers (e.g., following the COSMIN reporting guideline for patient-reported outcome measures (Gagnier et al., 2021)) could further enhance transparency by prompting researchers to provide information on all relevant aspects, including those not addressed, thereby ensuring comprehensive methodological reporting.

Furthermore, the a priori registration of questionnaire development plans (e.g., via the American Psychological Association Preregistration for Quantitative Research in Psychology template, ClinicalTrials.gov, or other preregistration templates available via the Open Science Framework), including proposed psychometric validation steps, could help clarify whether qualitative studies were never conducted or simply not made publicly available.

Structural validity and internal consistency were also areas of concern. While the LGBIS (Cramer et al., 2018; Mohr & Kendra, 2011) and LGBIS-DE (Niepel et al., 2019) demonstrated sufficient structural validity, other instruments, such as the LGBT Minority Stress Measure (Outland, 2016) and MIHI (Flebus et al., 2012), had insufficient results. Furthermore, internal consistency, which depends on demonstrated unidimensionality, was rated indeterminate for many instruments due to their indeterminate or insufficient structural validity.

Reliability data were limited across the reviewed instruments. Only the LGBIS (Cramer et al., 2018; Mohr & Kendra, 2011) had low-quality evidence for indeterminate reliability. Without reliable measurements, it is difficult to assess true differences between individuals and groups, which limits their utility in both research and clinical settings. Moreover, insufficient reliability further undermines the reproducibility of findings, exacerbating the limitations in the interpretation and application of results.

In contrast, several instruments showed positive results for construct validity, indicating that they were related to other variables in line with theoretical expectations. However, the quality of evidence for this property ranged from low to high, highlighting the need for further validation for some instruments.

Following the COSMIN-modified GRADE approach, we downgraded the quality of evidence for indirectness for the LGIS (Mohr & Fassinger, 2000) and the LGBIS-DE (Niepel et al., 2019). Indirectness indicates that the available evidence does not fully correspond to the question of the review. Our review focused on LGBQ adults and included instruments that at least targeted both lesbian and gay adults. Although the LGIS met this inclusion criterion, it was neither developed nor validated in LGBQ samples. For the LGBIS-DE, while the original LGBIS (Mohr & Kendra, 2011) was developed for lesbian, gay, and bisexual individuals, the German validation study excluded bisexual participants. Because the evidence base for both instruments therefore only partially reflects our intended target population, we downgraded the quality of evidence by one level due to indirectness. Importantly, this downgrade concerns the applicability of the available evidence to our review question rather than necessarily constituting a critique of the instruments themselves.

### Future Directions

Many measurement instruments in this systematic review were developed from literature strongly shaped by Meyer’s minority stress model (Meyer, 2003, 2015). Notably, the LGIS (Mohr & Fassinger, 2000) was developed in earlier theoretical contexts and does not draw on Meyer’s model in the way later instruments do. At the same time, theoretical work has increasingly emphasized processes that are less readily reflected by Meyer’s minority stress model including structural and institutionalized oppression, intersectional social positioning, and strengths-based or resilience-focused pathways (Frost & Meyer, 2023; Parra & Hastings, 2018; Riggs & Treharne, 2017).

None of the instruments included in this systematic review were developed within the more recent or expanded frameworks proposed in the literature (Feinstein, 2020; Hatzenbuehler, 2009; Pachankis et al., 2008; Perrin et al., 2020; Rivas‐Koehl et al., 2023). This pattern should be interpreted with care. Although our search strategy incorporated broad population and measurement terms to identify instruments assessing sexual identity minority, it did not explicitly include keywords related to these emerging frameworks. Consequently, the instruments identified in this review may represent an earlier stage of conceptual development, preceding the integration of newer theoretical perspectives. The absence of such frameworks in measurement development reflects a broader challenge in the field: the slow translation of theoretical advancements into validated instruments. This gap may limit the instruments’ applicability to individuals with multiple intersecting marginalized identities (e.g., transgender, non-binary, racial/ethnic minority LGBQ individuals) and their ability to capture social processes like invisibility, vigilance, and decompensation (Riggs & Treharne, 2017; Thoma et al., 2021).

### Strengths and Limitations

This systematic review has notable strengths, including a comprehensive and systematic search strategy, strict adherence to the COSMIN methodology, and independent assessments by two reviewers, ensuring transparency and methodological rigor. However, several limitations must be acknowledged.

A key limitation concerns our exclusion criterion. We deliberately restricted the review to instruments that had been examined across more than one sexual minority subgroup and, specifically, across more than one gender group. This decision was intended to identify comparatively generic instruments with potential utility for future cross-group analyses, rather than instruments tailored to a single subgroup. The requirement that studies include at least both lesbian and gay adults served as a pragmatic indicator of such broader applicability. However, this decision necessarily involved a trade-off. By prioritizing breadth of prior validation, we may have excluded instruments that are more specific, and possibly more sensitive, to the minority stress experiences of particular subgroups, including bisexual individuals. This should not be interpreted as a judgment that subgroup-specific instruments are less psychometrically sound, less relevant, or less appropriate. Rather, it reflects the specific aim of the present review. Accordingly, our findings are most informative for identifying instruments with broader cross-group applicability, but less informative for determining which instruments may best capture subgroup-specific stress processes. The choice between generic and subgroup-specific instruments ultimately depends on the research question. Future reviews focusing explicitly on subgroup-specific instruments would therefore be an important complement to the present review.

Further, the application of the COSMIN risk of bias checklist for content validity (Terwee et al., 2018) proved challenging. While the checklist requires detailed documentation of concept elicitation, cognitive interviewing, and pilot testing, many instruments relied primarily on theoretical frameworks and literature reviews. Following the COSMIN manual, the ratings on content validity are therefore—with exception of the DHEQ (Balsam et al., 2013)—based on the rating of reviewers in the absence of externally available evidence. Although COSMIN criteria were rigorously applied, these ratings remain subjective, reflecting the personal assessment of two reviewers. In most cases, while instrument development appeared theoretically sound, combining literature reviews with factor analysis, no qualitative development studies were published. Furthermore, we examined the measurement instruments in terms of their ability to reflect minority stress, even though this may not have been their original measurement goal. Therefore, an assessment of content validity with regard to the intended measurement construct could yield different results.

Another limitation relates to the evaluation of construct validity using the COSMIN criterion, “Were the measurement properties of comparator instruments adequate?” (Mokkink et al., 2018), which might be hindered by space constraints in journal publications possibly preventing authors from providing comprehensive details on comparator tools. This issue notably impacted the LGIS (Mohr & Fassinger, 2000) where evidence quality was downgraded. Excluding this criterion would have resulted in a moderate evidence rating rather than low quality for construct validity.

A final limitation arises from our retrospective alignment of instrument constructs with the minority stress model as presented in Fig. 2 (as adapted by Testa et al. (2015)). While this mapping exercise might point to dimensions less prominent in current research, it also emphasized the continued lack of terminological precision in minority stress research. For example, unidimensional tools like the Daily Sexual Minority Stressors Scale (Braitman et al., 2021; Heron et al., 2018) were excluded from psychometric evaluation based on our criteria of multidimensionality, yet the question remains whether such measures actually reflect an overly simplified conceptualization of minority stress. Interestingly, in our communication with one study author, the value of a multidimensional approach to measuring minority stress was emphasized (J. Mohr, personal communication, October 8, 2024). Advancing terminological and conceptual clarity in the field will be crucial for ensuring that future instruments not only meet psychometric standards but also reflect the lived realities and complex social positioning of diverse LGBQ populations.

### Lessons Learned

1. Measurement instruments capture different and, in some cases, non-overlapping dimensions of minority stress. To improve comparability, we encourage instrument developers to be more explicit in specifying the target construct and its theoretical anchors.
2. Content validity as understood by the COSMIN group is understudied in the field of minority stress research.

### Conclusion

Overall, this review identified substantial evidence gaps in the psychometric evaluation of minority stress measurement instruments, particularly concerning content validity. Given that content validity is a prerequisite for further psychometric evaluation, this is a critical area that requires further attention.

The LGBIS (Mohr & Kendra, 2011) and LGBIS-DE (Niepel et al., 2019) stand out as the most psychometrically robust instruments, consistently performing well across key properties such as internal consistency, structural validity, and construct validity. These tools outperform the original LGIS (Mohr & Fassinger, 2000) and offer promising resources for assessing aspects of minority stress in both research and clinical settings.

Other instruments, such as the DHEQ (Balsam et al., 2013), MIHI (Flebus et al., 2012), GALOSI-F/E (Highlen et al., 2000) and especially the LGBT Minority Stress Measure (Outland, 2016), require further validation and refinement to ensure their psychometric soundness and broader applicability. Future research should focus on developing and validating instruments based on qualitative research to more effectively capture the lived experiences of minority stress.

## Supplementary Information

Below is the link to the electronic supplementary material.

Supplementary file1 (DOCX 81 KB)

Supplementary file2 (DOCX 137 KB)

## Data Availability

All data extracted and analyzed in this systematic review are included in the article. Detailed COSMIN risk of bias ratings and summary tables for each individual instrument are available from the corresponding author upon reasonable request.
