# Supplementary material for: Instruments Used to Measure Sexual Identity Minority Stress: A COSMIN Systematic Review
Source: Arch Sex Behav. 2026 Aug 28;55(6):2829–48. doi: 10.1007/s10508-026-03508-z (PMC13597606; doi:10.1007/s10508-026-03508-z)
Supplement: Supplementary file 2 [file 10508_2026_3508_MOESM2_ESM.docx]

# Supplement 2

# Selection of Measurement Instruments for Dimensions of Minority Stress: Excluded References by Unmet Inclusion Category

## Inclusion Categories:

A1: Psychometric studies.

A2: Population: Adult homosexual, bisexual and queer persons

A3: Constructs measured with the instruments: Discrimination, Rejection, Victimization, Stigmatization, Internalized Homophobia, Negative Future Expectations, Concealment, Social Support, and Pride

A4: Review of validation studies.

A5: Publication in English or German.

A6: Full text available.

A7: Unique publication (not a duplicate).

## A1

Adelman, M. (1990): Stigma, gay lifestyles, and adjustment to aging: a study of later-life gay men and lesbians. In J Homosex 20 (3-4), pp. 7–32.

Ahlers, C. J.; Schaefer, G. A. (2006): SPM, Sexualpraeferenz-Maennchen (PSYNDEX Tests Info), Sexual Preference Figure/zpid.

Authors, Various (2013): Posters. In Psychology, Community & Health 2 (2), pp. 259–303. DOI: 10.5964/pch.v2i2.74.

Baams, Laura; Grossman, Arnold H.; Russell, Stephen T. (2015): Minority Stress and Mechanisms of Risk for Depression and Suicidal Ideation among Lesbian, Gay, and Bisexual Youth. In Developmental Psychology 51 (5). DOI: 10.1037/a0038994.

Beischel, William J. (2023): Gender pleasure: The positive affective component of gender/sex.DP - 2023. In Dissertation Abstracts International: Section B: The Sciences and Engineering 84 (4-B), No Pagination Specified. Available online at https://deepblue.lib.umich.edu/bitstream/handle/2027.42/174393/wbeisch_1.pdf?sequence=1&isAllowed=y.

Branstrom, R.; Pachankis, J. E. (2018): Validating the syndemic threat surrounding sexual minority men's health in a population-based study. In Eur. J. Public Health 28, pp. 87–88.

Brennan, James M.; Dunham, Kinsie J.; Bowlen, Morgan; Davis, Kelly; Ji, Gabriella; Cochran, Bryan N. (2021): Inconcealable: A cognitive-behavioral model of concealment of gender and sexual identity and associations with physical and mental health. In Psychology of Sexual Orientation and Gender Diversity 8 (1), pp. 80–93. DOI: 10.1037/sgd0000424.

Brenner, Danyell L. (2019): Relationships among minority stress, individual positive coping, and mental health in individuals identifying as a sexual minority. In Dissertation Abstracts International: Section B: The Sciences and Engineering 80 (2-B(E)), No-Specified. Available online at http://ovidsp.ovid.com/ovidweb.cgi?T=JS&PAGE=reference&D=psyc16&NEWS=N&AN=2018-58622-035.

Breuer, H.; Weuffen, M. (1996): DP, Differenzierungsprobe (PSYNDEX Tests Review), Breuer-Weuffen Discrimination Test/journal.

Budge, S. L.; Guo, E.; Mauk, E.; Tebbe, E. A. (2021): The development of an observational coding scheme to assess transgender and nonbinary clients' reported minority stress experiences. In Psychotherapy 58 (2), pp. 288–300. DOI: 10.1037/pst0000368.

Burke, T. B. (1997): Assessing homosexual stress. In J Homosex 33 (2), pp. 83–99.

Cerqueira-Santos, Elder; Silva, Bruno Brito; Rodrigues, Hênio dos Santos; Santos, Lizandra dos (2016): Internalized homophobia and marital satisfaction among male and female homosexuals. In Contextos Clínicos 9 (2), pp. 148–158. Available online at https://www.proquest.com/scholarly-journals/internalized-homophobia-marital-satisfaction/docview/1883709650/se-2.

Chan, R. C. H. (2022): Effects of Minority Stress on Group Identification and Collective Action among Sexual Minorities: A Longitudinal Test of the Rejection-Identification Model. In Sex Roles 87 (5-6), pp. 237–250. DOI: 10.1007/s11199-022-01304-2.

Chan, R. C. H.; Huang, Y. T. (2021): A Minority Stress Response Model of Health Behaviors in Gay and Bisexual Men: Results from a Taiwanese Sample. In LGBT health 8(3), pp. 209–221. DOI: 10.1089/lgbt.2020.0194.

Chan, R. C. H.; Mak, W. W. S. (2021): Resistance as a form of resilience in sexual and gender minorities: Differential moderating roles of collective action on the discrimination-depression relationship among sexual minority men and women. In Soc Sci Med 280, p. 114056. DOI: 10.1016/j.socscimed.2021.114056.

Chavanduka, Tanaka M. D.; Gamarel, Kristi E.; Todd, Kieran P.; Stephenson, Rob (2020): Responses to the gender minority stress and resilience scales among transgender and nonbinary youth. In Journal of LGBT Youth, No-Specified. DOI: 10.1080/19361653.2020.1719257.

Costa, Angelo Brandelli; Machado, Wagner de Lara; Bandeira, Denise Ruschel; Nardi, Henrique Caetano (2020): Response to a systematic review of the psychometric properties of Composite LGBT Prejudice and Discrimination Scales. In J Homosex 67 (6), pp. 735–736. DOI: 10.1080/00918369.2018.1557956.

Cross, Emily J.; Muise, Amy; Hammond, Matthew D. (2021): Do scales measuring sexist attitudes have equivalent meaning for sexual minorities and majorities? [References]. In Sex Roles 85 (11-12), pp. 707–720. DOI: 10.1007/s11199-021-01248-z.

Davidson, K.; McLaren, S.; Jenkins, M.; Corboy, D.; Gibbs, P. M.; Molloy, M. (2017): Internalized Homonegativity, Sense of Belonging, and Depressive Symptoms Among Australian Gay Men. In J Homosex 64 (4), pp. 450–465. DOI: 10.1080/00918369.2016.1190215.

Decker, H.; Combs, R. M.; Noonan, E. J.; Black, C.; Weingartner, L. A. (2022): LGBTQ+ Microaggressions in Health Care: Piloting an Observation Framework in a Standardized Patient Assessment. In J Homosex, pp. 1–17. DOI: 10.1080/00918369.2022.2122367.

Del Sánchez-Fuentes, María Mar; Parra-Barrera, Sandra M.; Fernandes de Araújo, Ludgleydson; Pereira Monteiro, Renan (2022): Validation of the Attitude Scale to Lesbian, Gay, Bisexual and Transgender Old Age in a Colombian Sample. In Sexuality & Culture. DOI: 10.1007/s12119-022-09989-z.

Drabble, L. A.; Mericle, A. A.; Wootton, A. R.; Munroe, C.; Li, L.; Trocki, K. F.; Hughes, T. (2021): Measuring the impact of legal recognition of same-sex marriage among sexual minority women. In Journal of GLBT Family Studies 17 (4), pp. 371–392. DOI: 10.1080/1550428x.2021.1935382.

Drabble, Laurie A.; Mericle, Amy A.; Wootton, Angie R.; Munroe, Cat; Li, Libo; Trocki, Karen F.; Hughes, Tonda (2021): Measuring the impact of legal recognition of same-sex marriage among sexual minority women. In Journal of GLBT Family Studies, No-Specified. DOI: 10.1080/1550428X.2021.1935382.

Dubke, R. E.; Corrigan, P. W. (2021): Intersectionality, Gender, and the Self-Stigma of Mental Illness. In J. Soc. Clin. Psychol. 40 (2), pp. 145–152. DOI: 10.1521/jscp.2021.40.2.145.

Dunaway, Krystall E.; Morrow, Jennifer A.; Porter, Bryan E. (2012): Development and Validation of the Cultural Competence of Program Evaluators (CCPE) Self-Report Scale. In American Journal of Evaluation 33 (4). DOI: 10.1177/1098214012445280.

Edwards, Christopher J. (2020): Clinical utility of the Minority Stress Scale (see STS/Innerlife) among gender and sexual minority identified patients. In Dissertation Abstracts International Section A: Humanities and Social Sciences 81 (2-A), No-Specified. Available online at http://ovidsp.ovid.com/ovidweb.cgi?T=JS&PAGE=reference&D=psyc17&NEWS=N&AN=2019-68369-010.

Felipe, Lou Collette; Garrett-Walker, J. J.; Montagno, Michelle (2022): Monoracial and multiracial LGBTQ+ people: Comparing internalized heterosexism, perceptions of racism, and connection to LGBTQ+ communities. In Psychology of Sexual Orientation and Gender Diversity 9 (1), pp. 1–11. DOI: 10.1037/sgd0000440.

Fletcher, Anne C.; Russell, Stephen T. (2001): Incorporating issues of sexual orientation in the classroom: Challenges and solutions. In Family Relations: An Interdisciplinary Journal of Applied Family Studies 50 (1), pp. 34–40. DOI: 10.1111/j.1741-3729.2001.00034.x.

Freese, R.; Ott, M. Q.; Rood, B. A.; Reisner, S. L.; Pantalone, D. W. (2018): Distinct Coping Profiles Are Associated With Mental Health Differences in Transgender and Gender Nonconforming Adults. In J Clin Psychol 74 (1), pp. 136–146. DOI: 10.1002/jclp.22490.

Frost, D. M.; Fingerhut, A. W.; Meyer, I. H. (2022): Social change and relationship quality among sexual minority individuals: Does minority stress still matter? In J Marriage Fam 84 (3), pp. 920–933. DOI: 10.1111/jomf.12827.

Gaspar, M.; Marshall, Z.; Rodrigues, R.; Adam, B. D.; Brennan, D. J.; Hart, T. A.; Grace, D. (2021): Mental health and structural harm: a qualitative study of sexual minority men's experiences of mental healthcare in Toronto, Canada. In Culture, health & sexuality 23 (1), pp. 98–114. DOI: 10.1080/13691058.2019.1692074.

Graham, Louis F. (2011): Examining the psychosocial health of black sexual minority men. In Dissertation Abstracts International: Section B: The Sciences and Engineering 71 (9-B), p. 5417. Available online at http://ovidsp.ovid.com/ovidweb.cgi?T=JS&PAGE=reference&D=psyc8&NEWS=N&AN=2011-99060-505.

Graham, S. M.; Mugo, P.; Gichuru, E.; Thiong'o, A.; Macharia, M.; Okuku, H. S. et al. (2013): Adherence to antiretroviral therapy and clinical outcomes among young adults reporting high-risk sexual behavior, including men who have sex with men, in coastal Kenya. In Aids Behav 17 (4), pp. 1255–1265. DOI: 10.1007/s10461-013-0445-9.

Graybill, Emily (2012): Social justice advocacy trends related to gay/ straight alliance advisors' experiences in schools. In Dissertation Abstracts International: Section B: The Sciences and Engineering 72 (11-B), p. 7081. Available online at http://ovidsp.ovid.com/ovidweb.cgi?T=JS&PAGE=reference&D=psyc9&NEWS=N&AN=2012-99100-487.

GREENE, B. (1994): Ethnic-minority lesbians and gay men : mental health and treatment issues : Mental health of lesbians and gay men. In J. consult. clin. psychol 62 (2), pp. 243–251.

Harper, Gary W.; Jamil, Omar Bashir; Wilson, Bianca D. M. (2007): Collaborative community-based research as activism: Giving voice and hope to lesbian, gay, and bisexual youth. In Journal of Gay & Lesbian Psychotherapy 11 (3-4), pp. 99–119. DOI: 10.1300/J236v11n03_06.

Horrillo, L.; Castro, A.; Matia, B.; Molina, L.; Garcia-Martinez, J.; Jaqueti, J. et al. (2019): Clinical aspects of visceral leishmaniasis caused by L. infantum in adults. Ten years of experience of the largest outbreak in Europe: what have we learned? In Parasit Vectors 12 (1), p. 359. DOI: 10.1186/s13071-019-3628-z.

indicated, No authorship (2018): "Multiple minority stress and LGBT community resilience among sexual minority men": Correction to McConnell et al. (2018). In Psychology of Sexual Orientation and Gender Diversity 5 (2), p. 168. DOI: 10.1037/sgd0000290.

Kamke, Kristyn; Widman, Laura; Javidi, Hannah (2022): The multidimensionality of adolescent girls' gender attitudes. In Gender Issues 39 (2), pp. 236–251. DOI: 10.1007/s12147-021-09288-1.

Keenan, K.; Berona, J.; Hipwell, A. E.; Stepp, S. D.; Romito, M. T. (2021): Validity of the Trier Social Stress Test in studying discrimination stress. In Stress 24 (1), pp. 113–119. DOI: 10.1080/10253890.2020.1741545.

Kinsey, Ginger M. (2003): Establishing the construct and discriminant validities of the hostile environment measure (hem). In Dissertation Abstracts International: Section B: The Sciences and Engineering 64 (1-B), p. 464. Available online at http://ovidsp.ovid.com/ovidweb.cgi?T=JS&PAGE=reference&D=psyc4&NEWS=N&AN=2003-95014-058.

Lee, J. I.; Chang, Y. P.; Tsai, C. S.; Yen, C. F. (2022): Internalized Sexual Stigma among Lesbian, Gay, and Bisexual Individuals in Taiwan: Its Related Factors and Association with Mental Health Problems. In International Journal of Environmental Research & Public Health [Electronic Resource] 19 (4), p. 19. Available online at https://ovidsp.ovid.com/ovidweb.cgi?T=JS&CSC=Y&NEWS=N&PAGE=fulltext&D=med20&AN=35206614 https://mdpi-res.com/d_attachment/ijerph/ijerph-14-00156/article_deploy/ijerph-14-00156.pdf?version=1486465342.

Liao, K. Y. H.; Kashubeck-West, S.; Weng, C. Y.; Deitz, C. (2015): Testing a Mediation Framework for the Link Between Perceived Discrimination and Psychological Distress Among Sexual Minority Individuals. In J. Couns. Psychol. 62 (2), pp. 226–241. DOI: 10.1037/cou0000064.

Lin, H. C.; Chang, C. C.; Chang, Y. P.; Chen, Y. L.; Yen, C. F. (2022): Associations among Perceived Sexual Stigma from Family and Peers, Internalized Homonegativity, Loneliness, Depression, and Anxiety among Gay and Bisexual Men in Taiwan. In International Journal of Environmental Research & Public Health [Electronic Resource] 19 (10), p. 20. Available online at https://ovidsp.ovid.com/ovidweb.cgi?T=JS&CSC=Y&NEWS=N&PAGE=fulltext&D=medl&AN=35627760 https://mdpi-res.com/d_attachment/ijerph/ijerph-14-00156/article_deploy/ijerph-14-00156.pdf?version=1486465342.

Mesa, D. A. (2022): USING VALIDATED INSTRUMENTS TO ASSESS LONELINESS, SOCIAL SUPPORT, AND BURNOUT AMONGST RACIAL AND LGBTQ plus RESIDENTS. In Journal of Investigative Medicine 70 (1), p. 159. DOI: 10.1136/jim-2022-WRMC.

Meyer, Ilan H. (2003): Prejudice, social stress, and mental health in lesbian, gay, and bisexual populations: Conceptual issues and research evidence. In Psychol. bull 129 (5), pp. 674–697. Available online at https://www.ncbi.nlm.nih.gov/pmc/articles/PMC2072932/pdf/nihms32623.pdf.

Salfas, B.; Rendina, H. J.; Parsons, J. T. (2019): What is the Role of the Community? Examining Minority Stress Processes among Gay and Bisexual Men. In Stigma health 4 (3), pp. 300–309. DOI: 10.1037/sah0000143.

Salim, S.; Robinson, M.; Flanders, C. E. (2019): Bisexual Women's Experiences of Microaggressions and Microaffirmations and Their Relation to Mental Health. In Psychology of Sexual Orientation and Gender Diversity 6 (3), pp. 336–346. DOI: 10.1037/sgd0000329.

Sarno, E. L.; Swann, G.; Newcomb, M. E.; Whitton, S. W. (2021): Intersectional Minority Stress and Identity Conflict Among Sexual and Gender Minority People of Color Assigned Female at Birth. In Cult. Divers. Ethn. Minor. Psychol. 27 (3), pp. 408–417. DOI: 10.1037/cdp0000412.

Scandurra, Cristiano; Monaco, Salvatore; Dolce, Pasquale; Nothdurfter, Urban (2020): Heteronormativity in italy: Psychometric characteristics of the italian version of the heteronormative attitudes and beliefs scale. In Sexuality Research & Social Policy: A Journal of the NSRC, No-Specified. DOI: 10.1007/s13178-020-00487-1.

Shangani, S.; Naanyu, V.; Operario, D.; Genberg, B. (2018): Stigma and Healthcare-Seeking Practices of Men Who Have Sex with Men in Western Kenya: A Mixed-Methods Approach for Scale Validation. In AIDS Patient Care STDS 32 (11), pp. 477–486. DOI: 10.1089/apc.2018.0101.

Shenkman, Geva; Shmotkin, Dov (2016): The hostile-world scenario and mental health concomitants among gays and lesbians. In Journal of Gay & Lesbian Mental Health 20 (1), pp. 70–86. DOI: 10.1080/19359705.2015.1048915.

Smallwood, Stacy W. (2014): Examining the relationships between religiosity, spirituality, internalized homonegativity, and sexual risk behaviors among African-American men who have sex with men: The Sexual Health in Faith Traditions (SHIFT) study. In Dissertation Abstracts International: Section B: The Sciences and Engineering 75 (1-B(E)), No-Specified. Available online at http://ovidsp.ovid.com/ovidweb.cgi?T=JS&PAGE=reference&D=psyc11&NEWS=N&AN=2014-99141-151.

Soulliard, Zachary A.; Vander Wal, Jillon S. (2020): Confirmatory factor analyses of the body image-acceptance and action questionnaire and functionality appreciation scale among lgbq adults. In Current Psychology: A Journal for Diverse Perspectives on Diverse Psychological Issues, No-Specified. DOI: 10.1007/s12144-020-01199-w.

Steimann, L.; Missala, I.; van-Kaick, S.; Walston, J. D.; Malzahn, U.; Heuschmann, P. U. et al. (2011): RASP DT, Rivermead Assessment of Somatosensory Performance - deutsche Fassung (PSYNDEX Tests Info), Rivermead Assessment of Somatosensory Performance (Winward, C. E., Halligan, P. W. & Wade, D. T., 2002) - German version/author.

Steinman, L.; Parrish, A.; Mayotte, C.; Bravo Acevedo, P.; Torres, E.; Markova, M. et al. (2021): Increasing Social Connectedness for Underserved Older Adults Living With Depression: A Pre-Post Evaluation of PEARLS. In American Journal of Geriatric Psychiatry 29(8), pp. 828–842. DOI: 10.1016/j.jagp.2020.10.005.

Stevens, Sally; Haverly, Katie; Powell, Claudia (2020): Improvements in self-acceptance for LGBTQ+ and straight allied youth and young adults enrolled in an affirming system of care program. In Children and Youth Services Review 118. DOI: 10.1016/j.childyouth.2020.105382.

Stewart, M. W. (2019): Health of Lesbian, Gay, Bisexual, Transgender, and Questioning Individuals. In J Perianesth Nurs 34 (5), pp. 1074–1076. DOI: 10.1016/j.jopan.2019.06.011.

Stojanovski, Kristefer; Zhou, Sasha; King, Elizabeth; Gjorgjiovska, Jovana; Mihajlov, Antonio (2018): "An application of the minority stress model in a non-Western context: Discrimination and mental health among sexual and gender minorities in Macedonia": Correction. In Sexuality Research & Social Policy: A Journal of the NSRC 15 (3), p. 377. DOI: 10.1007/s13178-017-0309-6.

Szymanski, Dawn M. (2004): Relations Among Dimensions of Feminism and Internalized Heterosexism in Lesbians and Bisexual Women. In Sex Roles 51 (3-4), pp. 145–159. DOI: 10.1023/B:SERS.0000037759.33014.55.

Szymanski, Dawn M.; Owens, Gina P. (2008): Do Coping Styles Moderate or Mediate the Relationship between Internalized Heterosexism and Sexual Minority Women's Psychological Distress? In Psychology of Women Quarterly 32 (1). DOI: 10.1111/j.1471-6402.2007.00410.x.

Talley, A. E.; Stevens, J. E. (2017): Sexual Orientation Self-Concept Ambiguity: Scale Adaptation and Validation. In Assessment 24 (5), pp. 632–645. DOI: 10.1177/1073191115617016.

Tan, S. M.; Cen, P.; Fang, T.; Yang, X.; Zhang, Y.; Zhu, J. W. et al. (2022): Chained multimediator model of sexual orientation disclosure, sexual minority stigma, sexual minority identity, social support, and resilience among ymsms. In BMC public health 22 (1). DOI: 10.1186/s12889-022-13231-8.

Tebbe, E. A.; Moradi, B.; Wilson, E.; Bell, H. L.; Connelly, K.; Lenzen, A. (2021): Development and psychometric evaluation of the Sexual Minority Women's Sexual Objectification Experiences Scale. In J. Couns. Psychol. 68(5), pp. 501–514. DOI: 10.1037/cou0000547.

Thaker, J.; Dutta, M.; Nair, V.; Rao, V. P. (2018): The Interplay between Stigma, Collective Efficacy, and Advocacy Communication among Men Who Have Sex with Men and Transgender Females. In J Health Commun 23 (7), pp. 614–623. DOI: 10.1080/10810730.2018.1499833.

Thoma, B. C.; Eckstrand, K. L.; Montano, G. T.; Rezeppa, T. L.; Marshal, M. P. (2021): Gender Nonconformity and Minority Stress Among Lesbian, Gay, and Bisexual Individuals: A Meta-Analytic Review. In Perspect. Psychol. Sci. 16 (6), pp. 1165–1183. DOI: 10.1177/1745691620968766.

Thomas, Allen D. (2017): The relationship of identity, discrimination, and life-satisfaction for Black LGBPQ individuals. In Dissertation Abstracts International: Section B: The Sciences and Engineering 77 (7-B(E)), No-Specified. Available online at http://ovidsp.ovid.com/ovidweb.cgi?T=JS&PAGE=reference&D=psyc14&NEWS=N&AN=2016-47711-134.

Thomas, B.; Mimiaga, M. J.; Mayer, K. H.; Perry, N. S.; Swaminathan, S.; Safren, S. A. (2012): The influence of stigma on HIV risk behavior among men who have sex with men in Chennai, India. In AIDS Care 24 (11), pp. 1401–1406. DOI: 10.1080/09540121.2012.672717.

Wade, Ryan M.; Harper, Gary W. (2020): Toward a multidimensional construct of racialized sexual discrimination (RSD): Implications for scale development. In Psychology of Sexual Orientation and Gender Diversity, No-Specified. DOI: 10.1037/sgd0000443.

Wade, Ryan M.; Harper, Gary W. (2021): Toward a multidimensional construct of racialized sexual discrimination (RSD): Implications for scale development. In Psychology of Sexual Orientation and Gender Diversity 8 (4), pp. 401–406. DOI: 10.1037/sgd0000443.

Waldo, C. R. (1999): Working in a majority context: A structural model of heterosexism as minority stress in the workplace. In J. Couns. Psychol. 46 (2), pp. 218–232. DOI: 10.1037/0022-0167.46.2.218.

Weiss, B. J.; Hope, D. A. (2011): A preliminary investigation of worry content in sexual minorities. In Journal of anxiety disorders 25 (2), pp. 244–250. DOI: 10.1016/j.janxdis.2010.09.009.

Wilkerson, J. M.; Smolenski, D. J.; Brady, S. S.; Rosser, B. R. (2012): Religiosity, Internalized Homonegativity, and Outness in Christian Men Who Have Sex with Men. In Sex Relationship Ther 27 (2), pp. 122–132. Available online at https://ovidsp.ovid.com/ovidweb.cgi?T=JS&CSC=Y&NEWS=N&PAGE=fulltext&D=pmnm2&AN=23226970.

Wilkerson, J. M.; Smolenski, D. J.; Brady, S. S.; Rosser, B. R. S. (2012): Religiosity, internalized homonegativity and outness in Christian men who have sex with men. In Sex Relationship Ther 27 (2), pp. 122–132. DOI: 10.1080/14681994.2012.698259.

Williams, M. T.; Wetterneck, C.; Tellawi, G.; Duque, G. (2015): Domains of distress among people with sexual orientation obsessions. In Archives of sexual behavior 44 (3), pp. 783–789. DOI: 10.1007/s10508-014-0421-0.

Young, W. J.; Bover Manderski, M. T.; Ganz, O.; Delnevo, C. D.; Hrywna, M. (2021): Examining the Impact of Question Construction on Reporting of Sexual Identity: Survey Experiment Among Young Adults. In JMIR Public Health Surveill 7 (12), e32294. DOI: 10.2196/32294.

Frei, Lukas.; Regli, Daniel.; Widmer, Adrian.; Znoj, Hansjörg. (2013): Angst vor Ablehnung als Risikofaktor für Suizidalität bei homo- und bisexuellen Personen. In Sexuologie - Zeitschrift für Sexualmedizin, Sexualtherapie und Sexualwissenschaft 20 (3-4), pp. 163–174. Available online at http://www.sexuologie-info.de/pdf/Bd.20_2013_2_1.pdf.

Frei, L.; Regli, D.; Widmer, A.; Znoj, H. (2012): LGBIS, Lesbian, Gay, and Bisexual Identity Scale - deutsche Fassung (PSYNDEX Tests Info), Lesbian, Gay, and Bisexual Identity Scale - German version/author.

No authorship, indicated (2022): "The Self-Acceptance of Sexuality Inventory (SASI): Development and validation": Correction to Camp, Vitoratou, and Rimes (2020). In Psychology of Sexual Orientation and Gender Diversity 9 (1), p. 20. DOI: 10.1037/sgd0000573.

Sattler, F. A.; Lemke, R. (2019): Testing the Cross-Cultural Robustness of the Minority Stress Model in Gay and Bisexual Men. In J Homosex 66 (2), pp. 189–208. DOI: 10.1080/00918369.2017.1400310.

Szymanski, Dawn M.; Chung, Y. Barry; Balsam, Kimberly F. (2001): Psychosocial correlates of internalized homophobia in lesbians. In Measurement and Evaluation in Counseling and Development 34 (1), pp. 27–38. Available online at http://ovidsp.ovid.com/ovidweb.cgi?T=JS&PAGE=reference&D=psyc3&NEWS=N&AN=2001-06272-003.

## A2

Anonymous (2019): "The Development and Psychometric Evaluation of the Trans Discrimination Scale: TDS-21": Correction to Watson et al. (2018). In J Couns Psychol 66 (1), p. 29. DOI: 10.1037/cou0000331.

Arcieri, A. A.; DeLucia, L. R. (2022): Development of a Scale of Prejudice toward Bisexual and Transgender Individuals on the Basis of Ambiguity Intolerance. In Journal of Bisexuality 22 (1), pp. 1–29. DOI: 10.1080/15299716.2022.2044959.

Basar, K.; Oz, G. (2016): [Resilience in Individuals with Gender Dysphoria: Association with Perceived Social Support and Discrimination]. In *Turk Psikiyatri Derg* 27 (4), pp. 225–234. Available online at https://ovidsp.ovid.com/ovidweb.cgi?T=JS&CSC=Y&NEWS=N&PAGE=fulltext&D=med13&AN=28046191.

Becker, C.; Schaefer, G. A.; Ahlers, C. J. (2007): FSL, Fragebogen zu sexualitaetsbezogenem Leidensdruck (PSYNDEX Tests Info), Sexual Distress Questionnaire/zpid. DOI: Tests.

Bedard-Thomas, Katherine K.; McKenna, John L.; Pantalone, David W.; Fireman, Gary; Marks, Amy K. (2019): A mixed-methods measurement study of female adolescent sexuality stress and support. In Psychology & Sexuality 10 (3), pp. 212–233. DOI: 10.1080/19419899.2019.1596972.

Bekker, M. H. J. (1993): The Development of an Autonomy Scale Based on Recent Insights into Gender Identity. In Eur. J. Personal. 7 (3), pp. 177–194. DOI: 10.1002/per.2410070304.

Berger, K.; Broll, S.; Winkelmann, J.; Heberlein, I.; Mueller, T.; Ries, V. (1999): PDQ-39, Parkinson's Disease Questionnaire - deutsche Fassung (PSYNDEX Tests Info), Parkinson's Disease Questionnaire (PDQ; Peto, V., Jenkinson, C., Fitzpatrick, R. & Greenhall, R., 1995) - German version/author, Synonym(e): Fragebogen zur Erfassung der Lebensqualitaet von Parkinson-Patienten. DOI: Stefan.

Billard, T. J. (2018): Attitudes Toward Transgender Men and Women: Development and Validation of a New Measure. In Front Psychol 9, p. 387. DOI: 10.3389/fpsyg.2018.00387.

Bockting, W. O.; Miner, M. H.; Swinburne Romine, R. E.; Dolezal, C.; Robinson, B. B. E.; Rosser, B. R. S.; Coleman, E. (2020): The Transgender Identity Survey: A Measure of Internalized Transphobia. In LGBT health 7 (1), pp. 15–27. DOI: 10.1089/lgbt.2018.0265.

Brenk, K. (2005): FSGSV, Fragebogen zur Sexualgeschichte und zum sexuellen Verhalten (PSYNDEX Tests Info), Sexual History and Sexual Behavior Questionnaire/zpid. Available online at https://ir.ua.edu/handle/123456789/3139.

Brewster, Melanie E.; Esposito, Jessica (2017): Chronic Illness Rejection and Discrimination Scale: An instrument modification and confirmatory factor analysis. In Stigma and Health 2 (1), pp. 16–22. DOI: 10.1037/sah0000039.

Brown, Michael J.; Henriquez, Ernesto (2011): Support for gay and lesbian civil rights: Development and examination of a new scale. In J Homosex 58 (4), pp. 462–475. DOI: 10.1080/00918369.2011.555664.

Cardenas, Manuel; Barrientos, Jaime Eduardo (2008): The attitudes toward lesbians and gay men scale (ATLG): Adaptation and testing the reliability and validity in Chile. In Journal of sex research 45 (2), pp. 140–149. DOI: 10.1080/00224490801987424.

Carrera-Fernandez, M. V.; Lameiras-Fernandez, M.; Rodriguez-Castro, Y.; Vallejo-Medina, P. (2014): Spanish adolescents' attitudes toward transpeople: proposal and validation of a short form of the Genderism and Transphobia Scale. In Journal of sex research 51 (6), pp. 654–666. DOI: 10.1080/00224499.2013.773577.

Catalpa, J. M.; McGuire, J. K.; Fish, J. N.; Nic Rider, G.; Bradford, N.; Berg, D. (2019): Predictive validity of the genderqueer identity scale (GQI): differences between genderqueer, transgender and cisgender sexual minority individuals. In Int J Transgend 20 (2-3), pp. 305–314. DOI: 10.1080/15532739.2018.1528196.

Cohen, Joshua Marcus (2001): An investigation of stigma and shame in HIV and homosexuality: An affect theory and shame theory perspective. (immune deficiency). In Dissertation Abstracts International: Section B: The Sciences and Engineering 62 (1-B), p. 540. DOI: Health.

Cullen, Claire (2021): Developing the Implicit Relational Assessment Procedure (IRAP) as a measure of implicit homonegativity. In Dissertation Abstracts International: Section B: The Sciences and Engineering 82 (7-B), No-Specified. DOI: 3100.

Daly, Jillian (1991): Measuring attitudes toward lesbians and gay men: Development and initial psychometric evaluation of an instrument. In Dissertation Abstracts International: Section B: The Sciences and Engineering 52 (5-B), p. 2816. DOI: Scales.

Del Sanchez-Fuentes, Maria Mar; Parra-Barrera, Sandra M.; Fernandes de Araujo, Ludgleydson; Pereira Monteiro, Renan (2022): Validation of the Attitude Scale to Lesbian, Gay, Bisexual and Transgender Old Age in a Colombian sample. In Sexuality & Culture 26 (6), pp. 2138–2150. DOI: 10.1007/s12119-022-09989-z.

Delong, G.; Kim, D.; Kiperman, S. (2023): On Outness: Validity Evidence of the Outness Inventory for Sexual and Gender Minoritized Adolescents. In - Youth Soc. DOI: 10.1177/0044118X231216750.

Dillon, F. R.; Alessi, E. J.; Craig, S.; Eber-Sole, R. C.; Kumar, S. M.; Spadola, C. (2015): Development of the Lesbian, Gay, and Bisexual Affirmative Counseling Self-Efficacy Inventory - Short Form (LGB-CSI-SF). In Psychology of Sexual Orientation and Gender Diversity 2 (1), pp. 86–95. Available online at https://ovidsp.ovid.com/ovidweb.cgi?T=JS&CSC=Y&NEWS=N&PAGE=fulltext&D=pmnm3&AN=25992391https://www.ncbi.nlm.nih.gov/pmc/articles/PMC4433756/pdf/nihms686800.pdf.

Duncan, L. E.; Mincer, E.; Dunn, S. R. (2017): Assessing Politicized Sexual Orientation Identity: Validating the Queer Consciousness Scale. In J Homosex 64 (8), pp. 1069–1091. DOI: 10.1080/00918369.2016.1236599.

Edwards, Lindsay L.; Hanley, Scotty M. (2021): Scale of internalized trans oppression: Measure development and exploratory factor analysis. In Contemporary Family Therapy: An International Journal, No-Specified. DOI: 10.1007/s10591-020-09564-4.

Foster, Aasha Brianna (2018): Measuring social invisibility and erasure: Development of the Asexual Microaggressions Scale. In Dissertation Abstracts International: Section B: The Sciences and Engineering 79 (4-B(E)), No-Specified. DOI: 2100.

Freitas, Daniela Fonseca; Coimbra, Susana; Marturano, Edna Maria; Fontaine, Anne Marie (2015): Adaptation of the Everyday Discrimination scale to Portuguese youth. In Adaptacao da Escala de Discriminacao Quotidiana para Jovens Portugueses 28 (4), pp. 708–717. DOI: 10.1590/1678-7153.201528408.

Green, Jane (2024): Development, reliability, and validity of a measurement of attitudes toward non-binary individuals.DP - 2024. In Dissertation Abstracts International Section A: Humanities and Social Sciences 85 (3-A). DOI: Study.

Jardin, C.; Garey, L.; Zvolensky, M. J. (2017): Measuring Sexual Motives: A Test of the Psychometric Properties of the Sexual Motivations Scale. In Journal of sex research 54 (9), pp. 1209–1219. DOI: 10.1080/00224499.2016.1238031.

Keum, B. T.; Miller, M. J. (2018): Measurement invariance of the perceived online racism scale across age and gender. In Cyberpsychology 12 (3). DOI: 10.5817/CP2018-3-3.

Lee, Sang Jung; Gopalan, Geetha; Harrington, Donna (2016): Validation of the Parenting Stress Index--Short Form with Minority Caregivers. In Research on Social Work Practice 26 (4). DOI: 10.1177/1049731514554854.

Levant, Ronald; Pryor, Shana; Silver, Kristin (2022): The woman's nontraditional sexuality questionnaire-short form (WNSQ-SF): Development, variance composition, reliability, validity, and measurement invariance. In Gender Issues 39 (4), pp. 409–436. DOI: 10.1007/s12147-022-09298-7.

Matsuda, Win T.; Rouse, Steven V.; Miller-Perrin, Cindy L. (2014): Validation of the Attitudes Regarding Bisexuality Scale: Correlations with ratings of a positive media image of bisexual men and women. In Journal of Bisexuality 14 (2), pp. 265–276. DOI: 10.1080/15299716.2014.903219.

Molero, Fernando; Recio, Patricia; Garcia-Ael, Cristina; Fuster, Maria J.; Sanjuan, Pilar (2013): Measuring dimensions of perceived discrimination in five stigmatized groups. In Social Indicators Research 114 (3), pp. 901–914. DOI: 10.1007/s11205-012-0179-5.

Morrison, M. A.; Morrison, T. G. (2000): The construction and validation of a scale measuring 'modern' prejudice toward gay men and lesbians. In Int. J. Psychol. 35 (3-4), p. 105. DOI: scale.

Orphanidys, Jaymie Campbell (2019): To the T: Reliability of the LGBT-POC Microaggressions Scale. In Dissertation Abstracts International Section A: Humanities and Social Sciences 80 (1-A(E)), No-Specified. DOI: Social.

Prati, Gabriele (2012): Development and psychometric properties of the Homophobic Bullying Scale. In Educational and Psychological Measurement 72 (4), pp. 649–664. DOI: 10.1177/0013164412440169.

Pratt-Chapman, M. L.; Ward, A. R. (2022): The Index of Attitudes Toward Homosexuals: Factor Analysis in a National Sample of Oncology Clinicians. In J Homosex 69(7), pp. 1238–1251. DOI: 10.1080/00918369.2021.1909394.

Ray, Travis N.; Parkhill, Michele R. (2021): Heteronormativity, disgust sensitivity, and hostile attitudes toward gay men: Potential mechanisms to maintain social hierarchies. In Sex Roles 84 (1-2), pp. 49–60. DOI: 10.1007/s11199-020-01146-w.

Robinson, Matthew A. (2015): The development of the lesbian, gay, bisexual affiliate stigma measure. In Dissertation Abstracts International: Section B: The Sciences and Engineering 75 (11-B(E)), No-Specified. DOI: Health.

Robinson, M. A.; Brewster, M. E. (2016): Understanding affiliate stigma faced by heterosexual family and friends of LGB people: A measurement development study. In J Fam Psychol 30 (3), pp. 353–363. DOI: 10.1037/fam0000153.

Rodriguez-Castro, Yolanda; Lameiras-Fernandez, Maria; Carrera-Fernandez, Victoria; Vallejo-Medina, Pablo (2013): Validation of the Modern Homophobia Scale in a sample of adolescents. In Validacion de la Escala de Homofobia Moderna en una muestra de adolescentes 29 (2), pp. 523–533. DOI: Scales.

Rossiter, William T. (1980): Homophobia: A construct validity study of MacDonald's Attitudes Toward Homosexuality Scale-Male. In Dissertation Abstracts International: Section B: The Sciences and Engineering 41 (6-B), p. 2344. DOI: Tests.

Scandurra, Cristiano; Bochicchio, Vincenzo; Dolce, Pasquale; Carava, Chiara; Vitelli, Roberto; Testa, Rylan Jay; Balsam, Kimberly F. (2020): The Italian validation of the gender minority stress and resilience measure. In Psychology of Sexual Orientation and Gender Diversity 7 (2), pp. 208–221. DOI: 10.1037/sgd0000366.

Scheim, Ayden I. (2023): Discrimination and health: Measurement and impacts on Ontario's transgender communities.DP - 2023. In Dissertation Abstracts International: Section B: The Sciences and Engineering 84 (3-B), No Pagination Specified. DOI: Study.

Schulze, B.; Stuart, H.; Riedel-Heller, S. G. (2008): ISE, Inventar Subjektiver Stigmaerfahrungen (PSYNDEX Tests Info), Inventory of Stigmatising Experiences (ISE) (ISE; Stuart, H., Milev, R. & Koller, M., 2005) - German version/author. DOI: Tests.

Scierri, Irene Dora Maria; Batini, Federico (2020): Measuring homonegativity: Italian validation of the Multidimensional Scale of Attitudes Toward Lesbians and Gay Men. In Misurare l'omonegativita: validazione italiana della Multidimensional Scale of Attitudes Toward Lesbians and Gay Men 21, pp. 169–190. DOI: Tests.

Sibitz, I.; Friedrich, M. E.; Unger, A.; Bachmann, A.; Benesch, T.; Amering, M. (2013): ISMI, Internalized Stigma of Mental Illness-Skala - deutsche Fassung (PSYNDEX Tests Info), Internalized Stigma of Mental Illness Scale (ISMI; Ritsher, J.B., Otilingam, P.G. & Grajales, M., 2003) - German version/author. DOI: stigma.

Strickland, Noelle J.; Nogueira-Arjona, Raquel; Mackinnon, Sean; Wekerle, Christine; Stewart, Sherry H. (2021): Clarifying the factor structure of the Self-Compassion Scale: Nested comparisons of six confirmatory factor analysis models. In European Journal of Psychological Assessment (Pagination). DOI: 10.1027/1015-5759/a000672.

Swann, G.; Minshew, R.; Newcomb, M. E.; Mustanski, B. (2016): Validation of the Sexual Orientation Microaggression Inventory in Two Diverse Samples of LGBTQ Youth. In Arch Sex Behav 45 (6), pp. 1289–1298. DOI: 10.1007/s10508-016-0718-2.

Testa, Rylan J.; Habarth, Janice; Peta, Jayme; Balsam, Kimberly; Bockting, Walter (2015): Development of the Gender Minority Stress and Resilience Measure. In Psychology of Sexual Orientation and Gender Diversity 2 (1), pp. 65–77. DOI: 10.1037/sgd0000081.

Ungar, Michael; Liebenberg, Linda (2011): Assessing Resilience across Cultures Using Mixed Methods: Construction of the Child and Youth Resilience Measure. In Journal of Mixed Methods Research 5 (2). DOI: 10.1177/1558689811400607.

Warren, A. R.; Steffen, A. M. (2020): Development of a Transgender and Gender Nonconforming Language Self-Efficacy Scale for Social Service Providers Working With Older Adults. In J Appl Gerontol 39 (5), pp. 555–560. DOI: 10.1177/0733464818759754.

Weeks, S. N.; Renshaw, T. L.; Serang, S. (2023): Validating the Structure of Proximal and Distal Factors within the Sexual Minority Adolescent Stress Inventory. In - J. Psychoeduc. Assess. 41 (2), p. 152. DOI: 10.1177/0734282922113317.

Windsor, L. C.; Benoit, E.; Ream, G. L.; Forenza, B. (2013): The provider perception inventory: psychometrics of a scale designed to measure provider stigma about HIV, substance abuse, and MSM behavior. In AIDS Care 25 (5), pp. 586–591. DOI: 10.1080/09540121.2012.726338.

Worthington, Roger L.; Dillon, Frank R.; Becker-Schutte, Ann M. (2005): Development, Reliability, and Validity of the Lesbian, Gay, and Bisexual Knowledge and Attitudes Scale for Heterosexuals (LGB-KASH). In J Couns Psychol 52 (1), pp. 104–118. DOI: 10.1037/0022-0167.52.1.104.

Wright Jr, L. W.; Adams, H. E.; Bernat, J. (1999): Development and validation of the Homophobia Scale. In Journal of Psychopathology and Behavioral Assessment 21 (4), pp. 337–347. DOI: 10.1023/a:1022172816258.

Xia, Xiaoyang (2022): An alternative measure for attitudes toward bisexual people. In Psychology of Sexual Orientation and Gender Diversity 9 (3), pp. 321–342. DOI: 10.1037/sgd0000471.

Zarwell, M.; Walsh, J. L.; Quinn, K. G.; Kaniuka, A.; Patton, A.; Robinson, W. T.; Cramer, R. J. (2021): A psychometric assessment of a network social capital scale among sexual minority men and gender minority individuals. In BMC public health 21 (1). DOI: 10.1186/s12889-021-11970-8.

Zvolensky, M. J.; Shepherd, J. M.; Garey, L.; Woody, M.; Otto, M. W.; Clausen, B. et al. (2024): Negative emotional reactivity to minority stress: measure development and testing. In Cognitive Behav Ther 53 (1), pp. 1–28. DOI: 10.1080/16506073.2023.2260560.

Estrada, Armando X.; Probst, Tahira M.; Brown, Jeremiah; Graso, Maja (2011): Evaluating the psychometric and measurement characteristics of a measure of sexual orientation harassment. In Military Psychology 23 (2), pp. 220–236. DOI: 10.1080/08995605.2011.559394.

## A3

Algarin, Angel B.; Shrader, Cho Hee; Hackworth, Benjamin T.; Varas-Diaz, Nelson; Fennie, Kristopher P.; Sheehan, Diana M.; Ibanez, Gladys E. (2021): Development and validation of the Community PrEP-related Stigma Scale (Community-PSS). In AIDS Education and Prevention 33 (2), pp. 120–128. DOI: 10.1521/aeap.2021.33.2.120.

Alvis, Lauren M. (2020): Distinguishing beliefs about social inequality: Associations among dimensions of critical consciousness. In Dissertation Abstracts International Section A: Humanities and Social Sciences 81 (11-A), No-Specified. DOI: 2800.

Anderson, Joel R.; Koc, Yasin; Falomir-Pichastor, Juan Manuel (2018): The English version of the Attitudes Toward Homosexuality Scale. In Swiss Journal of Psychology 77 (3), pp. 117–126. DOI: 10.1024/1421-0185/a000210.

Anderson, R. E.; Namie, E. M. C.; Goodman, E. L. (2021): Valid for who? A preliminary investigation of the validity of two sexual victimization questionnaires in men and sexual minorities. In Am 46 (1), pp. 168–185. DOI: 10.1007/s12103-020-09589-3.

Arcieri, Amanda A.; DeLucia, Lacey Rose (2022): Development of a scale of prejudice toward bisexual and transgender individuals on the basis of ambiguity intolerance. In Journal of Bisexuality 22 (1), pp. 1–29. DOI: 10.1080/15299716.2022.2044959.

Arrington, R.; Cofrancesco, J.; Wu, A. W. (2004): Questionnaires to measure sexual quality of life. In Qual Life Res 13 (10), pp. 1643–1658. DOI: Review.

Arseneau, J. R.; Grzanka, P. R.; Miles, J. R.; Fassinger, R. E. (2013): Development and initial validation of The Sexual Orientation Beliefs Scale (SOBS). In J Couns Psychol 60 (3), pp. 407–420. DOI: 10.1037/a0032799.

Badenes-Ribera, L.; Frias-Navarro, D.; Monterde, I. Bort H.; Pascual-Soler, M. (2016): Spanish Validation of the Polymorphous Prejudice Scale in a Sample of University Students. In J Homosex 63 (11), pp. 1517–1536. DOI: 10.1080/00918369.2016.1223349.

Baiocco, M.; Blais, M.; Samoilenko, M.; Chamberland, L.; Côté, I. (2023): Development of a composite job quality index for LGBTQ plus workers in Quebec (Canada). In - Int. Labour Rev. 162 (2), pp. 327–353. DOI: 10.1111/ilr.12400.

Baker, N. A.; Halford, W. K. (2020): Assessment of Couple Relationships Standards in Same-Sex Attracted Adults. In Fam Process 59 (2), pp. 537–555. DOI: 10.1111/famp.12447.

Beck, Lisa (2018): A mixed-methods approach to developing a measure of attitudes toward the LGBTQIA+ community. In Dissertation Abstracts International Section A: Humanities and Social Sciences 78 (7-A(E)), No-Specified. DOI: Health.

Bidell, Markus P. (2019): "The Lesbian, Gay, Bisexual, and Transgender Development of Clinical Skills Scale (LGBT-DOCSS): Establishing a new interdisciplinary self-assessment for health providers": Correction. In J Homosex 66 (9), p. 1308. DOI: 10.1080/00918369.2019.1617602.

Blashill, A. J.; Wilson, J. M.; Baker, J. S.; Mayer, K. H.; Safren, S. A. (2014): Assessing appearance-related disturbances in HIV-infected men who have sex with men (MSM): psychometrics of the body change and distress questionnaire-short form (ABCD-SF). In Aids Behav 18 (6), pp. 1075–1084. DOI: 10.1007/s10461-013-0620-z.

Briere, J.; Runtz, M.; Rodd, K. (2024): Social maltreatment as trauma: Posttraumatic correlates of a new measure of exposure to sexism, racism, and cisheterosexism. In Psychol Trauma 22, p. 22. DOI: 10.1037/tra0001636.

Brownfield, Jenna M.; Flores, Mirella J.; Morgan, Sydney K.; Allen, Luke R.; Marszalek, Jacob M. (2018): Development and psychometric properties of the Evasive Attitudes of Sexual Orientation Scale (EASOS). In Psychology of Sexual Orientation and Gender Diversity 5 (1), pp. 44–56. DOI: 10.1037/sgd0000256.

Budhwani, H.; Yigit, I.; Maragh-Bass, A. C.; Rainer, C. B.; Claude, K.; Muessig, K. E.; Hightow-Weidman, L. B. (2022): Development and Validation of the Youth Pre-Exposure Prophylaxis (PrEP) Stigma Scale. In Aids Behav 27, p. 27. DOI: Using.

Bunting, S. R.; Chirica, M. G.; Ritchie, T. D.; Garber, S. S.; Batteson, T. J. (2021): A National Study of Medical Students' Attitudes Toward Sexual and Gender Minority Populations: Evaluating the Effects of Demographics and Training. In LGBT health 8 (1), pp. 79–87. DOI: 10.1089/lgbt.2020.0288.

Burke, Timothy Brian (1997): The effect of gay male sex role adherence on others' homosexual stress and attitudes toward gay men. In Dissertation Abstracts International: Section B: The Sciences and Engineering 57 (10-B), p. 6635. DOI: 3100.

Castillo-Mayen, Rosario; Vivas, Sebastian; Garcia-Torres, Francisco; Tabernero, Carmen; Santiago-Sillero, Sandra; Onieva-Caston, Laura; Luque, Barbara (2024): The gender-specific binegativity scale: Validation in a spanish sample of university students and proposal of a brief version. In Sex Res Soc Policy 21, pp. 279–296. DOI: 10.1007/s13178-023-00912-1.

Checa, Irene; Di Marco, Donatella; Bohorquez, M. (2022): Measurement invariance of the satisfaction with life scale by sexual orientation. In Archives of sexual behavior, No Pagination Specified. DOI: 10.1007/s10508-021-02240-0.

Chu, J.; Floyd, R.; Diep, H.; Pardo, S.; Goldblum, P.; Bongar, B. (2013): A tool for the culturally competent assessment of suicide: the Cultural Assessment of Risk for Suicide (CARS) measure. In Psychological assessment 25 (2), pp. 424–434. DOI: 10.1037/a0031264.

Convertino, A. D.; Gonzales, M. th; Malcarne, V. L.; Blashill, A. J. (2019): A psychometric investigation of the Sociocultural Attitudes Towards Appearance Questionnaire-4-Revised among sexual minority adults in the U.S. In Body image 31, pp. 96–101. DOI: 10.1016/j.bodyim.2019.08.013.

Costa, Angelo Brandelli; Bandeira, Denise Ruschel; Nardi, Henrique Caetano (2015): Evaluation of prejudice against sexual and gender diversity: Construction of an instrument. In Avaliacao do preconceito contra diversidade sexual e de genero: Construcao de um instrumento 32 (2), pp. 163–172. DOI: 10.1590/0103-166X2015000200002.

Dannecker, M. (1990): B-MHS, BEFRAGUNG UEBER MAeNNLICHE HOMOSEXUALITAeT (PSYNDEX Tests Review), Male Homosexuality Questionnaire/zpid. DOI: sich.

Day, N.; Meglich, P.; Porter, T. H. (2021): Measuring Bullying in Sexual Minorities: Testing Two Bullying Scales in an LGB Sample. In J Homosex, pp. 1–25. DOI: 10.1080/00918369.2021.1909393.

Del Pino, H. E.; Steers, W. N.; Lee, M.; McCuller, J.; Hays, R. D.; Harawa, N. T. (2021): Measuring Gender Role Conflict, Internalized Stigma, and Racial and Sexual Identity in Behaviorally Bisexual Black Men. In Archives of sexual behavior 16, p. 16. DOI: 10.1007/s10508-021-01925-w.

Dinkel, A.; Nather, C.; Jaeger, H.; Jaegel-Guedes, E.; Lahmann, C.; Steinke, C. et al. (2014): HSS-D, HIV-Stigma Skala (PSYNDEX Tests Info), HIV Stigma Scale (HSS; Berger, B.E., Ferrans, C.E. & Lashley, F.R., 2001) - German version/author, Synonym(e): HIV-Stigma-Skala, Varianten: HSS-D21.

Diogenes de Medeiros, E.; Fernandes de Araujo, L.; Victor de Oliveira Santos, J.; Souza, T. C.; Monteiro, R. P. (2019): Attitudes towards Lesbian, Gay, Bisexual and Transgender Old Age Scale (EAFV- LGBT): Elaboration and Psychometric Evidence. In The Spanish journal of psychology 22, E14. DOI: 10.1017/sjp.2019.14.

Elizondo, J. E.; Trevino, A. C.; Violant, D.; Rivas-Estilla, A. M.; Alvarez, M. M. (2018): [Men who have sex with men and human immunodeficiency virus testing in dental practice]. In Gac Sanit 32 (3), pp. 262–268. DOI: 10.1016/j.gaceta.2017.04.008.

Evans, Christopher A. (2023): Development and initial validation of the Sexual Minority Social Surrogate Scale.DP - 2023. In Dissertation Abstracts International Section A: Humanities and Social Sciences 84 (4-A), No Pagination Specified. DOI: Study.

Ghabrial, M. A.; Andersen, J. P. (2021): Development and initial validation of the Queer People of Color Identity Affirmation Scale. In J Couns Psychol 68 (1), pp. 38–53. DOI: 10.1037/cou0000443.

Gilbert, P. A.; Rhodes, S. D. (2012): Psychometric Performance of a Novel Measure of Social Support Among Spanish-Speaking Immigrant Latino Gay Men. In Hispanic journal of behavioral sciences 34 (3), pp. 491–504. DOI: 10.1177/0739986312446290.

Goldbach, Chloe; Knutson, Douglas (2021): Gender-related minority stress and gender dysphoria: Development and initial validation of the Gender Dysphoria Triggers Scale (GDTS). In Psychology of Sexual Orientation and Gender Diversity (Pagination). DOI: 10.1037/sgd0000548.

Goldbach, Chloe; Knutson, Douglas (2023): Gender-related minority stress and gender dysphoria: Development and initial validation of the Gender Dysphoria Triggers Scale (GDTS). In Psychology of Sexual Orientation and Gender Diversity 10 (3), pp. 383–396. DOI: 10.1037/sgd0000548.

Gruber, F. M. Distlberger E. Scherndl T. Ortner T. M. Pletzer B. (2020): GERAS Gender Related Attribute Survey (PSYNDEX Tests Info) Gender Related Attribute Survey (GERAS)/author Synonym(e): Assessment of Gender-Related Attributes (German version). In 2020 Psychometric properties of the multifaceted Gender-Related Attributes Survey (GERAS). European Journal of Psychological Assessment, 36 (4), 612-623. DOI: Other.

Guzman-Gonzalez, Monica; Garrido-Rojas, Lusmenia; Bahamondes, Joaquin; Gomez, Fabiola; Barrientos, Jaime; Espinoza-Tapia, Ricardo (2021): Psychometric properties of the Spanish version of the Emotional Intimacy Scale with heterosexual, gay and lesbian individuals in couple relationships. In Terapia Psicologica 39 (2), pp. 219–235. DOI: 10.4067/s0718-48082021000200219.

Han, Eunae; Hoffman, Lesa; Wood, Susannah M. (2023): Using the Internalized Misogyny Scale across sexual orientations: Measurement invariance and item factor analysis. In Sex Roles 88 (5-6), pp. 290–304. DOI: 10.1007/s11199-023-01357-x.

Hart, Trevor A.; Noor, Syed W.; Tulloch, Tyler G.; Sivagnanasunderam, Buvani; Vernon, Julia R. G.; Pantalone, David W. et al. (2019): The Gender Nonconformity Teasing Scale for gay and bisexual men. In Psychology of men & masculinity 20 (3), pp. 445–457. DOI: 10.1037/men0000179.

Hart, Trevor A.; Rotondi, Nooshin Khobzi; Souleymanov, Rusty; Brennan, David J. (2015): Psychometric properties of the Social Appearance Anxiety Scale among Canadian gay and bisexual men of color. In Psychology of Sexual Orientation and Gender Diversity 2 (4), pp. 470–481. DOI: 10.1037/sgd0000140.

Jackman, K. M. P.; Kane, J.; Kharrazi, H.; Johnson, R. M.; Latkin, C. (2021): Using the Patient Portal Sexual Health Instrument in Surveys and Patient Questionnaires Among Sexual Minority Men in the United States: Cross-sectional Psychometric Validation Study (vol 23, e18750, 2021). In J. Med. Internet Res. 23 (3). DOI: 10.2196/283580.

Kaniuka, Andrea R. (2023): Mental health promotion and suicide prevention among sexually and gender diverse adults.DP - 2023. In Dissertation Abstracts International Section A: Humanities and Social Sciences 84 (5-A), No Pagination Specified. DOI: Study.

Klassen, A. F.; Kaur, M.; Johnson, N.; Kreukels, B. P.; McEvenue, G.; Morrison, S. D. et al. (2018): International phase I study protocol to develop a patient-reported outcome measure for adolescents and adults receiving gender-affirming treatments (the GENDER-Q). In BMJ Open 8 (10), e025435. DOI: 10.1136/bmjopen-2018-025435.

Lampis, Jessica; Simone, Silvia de; Belous, Christopher K. (2021): Relationship satisfaction, social support, and psychological well-being in a sample of Italian lesbian and gay individuals. In Journal of GLBT Family Studies 17 (1), pp. 49–62. DOI: 10.1080/1550428X.2020.1724844.

Lehavot, Keren (2012): Minority stress, coping, and health among sexual minority women. In Dissertation Abstracts International: Section B: The Sciences and Engineering 73 (2-B), p. 1255. DOI: Health.

Lehavot, Keren; King, Kevin M.; Simoni, Jane M. (2011): Development and validation of a gender expression measure among sexual minority women. In Psychology of Women Quarterly 35 (3), pp. 381–400. DOI: 10.1177/0361684311413554.

Lin, C. Y.; Tsai, C. S.; Fan, C. W.; Griffiths, M. D.; Chang, C. C.; Yen, C. F.; Pakpour, A. H. (2022): Psychometric Evaluation of Three Versions of the UCLA Loneliness Scale (Full, Eight-Item, and Three-Item Versions) among Sexual Minority Men in Taiwan. In International Journal of Environmental Research & Public Health [Electronic Resource] 19 (13), p. 1. DOI: Using.

Lindley, Louis; Bauerband, Loren (2022): Measurement invariance of the Depression, Anxiety, and Stress Scale (DASS-21) across cisgender sexual minority and transgender and nonbinary individuals. In Psychology of Sexual Orientation and Gender Diversity (Pagination). DOI: 10.1037/sgd0000554.

Lindley, Louis; Budge, Stephanie L. (2022): Development and validation of the Trans and Nonbinary Coping Measure (TNCM): A measure of trans and nonbinary specific ways of coping with gender-related stress. In Psychology of Sexual Orientation and Gender Diversity, No Pagination Specified. DOI: 10.1037/sgd0000618.

Lopez-Saez, Miguel Angel; Garcia-Dauder, Dau; Montero, Ignacio; Lecuona, Oscar (2022): Adaptation and validation of the Evasive Attitudes of Sexual Orientation Scale into Spanish. In J Homosex 69 (5), pp. 925–945. DOI: 10.1080/00918369.2021.1898803.

Lopez-Saez, M. A.; Garcia-Dauder, D.; Montero, I.; Lecuona, O. (2022): Adaptation and Validation of the Evasive Attitudes of Sexual Orientation Scale into Spanish. In J Homosex 69(5), pp. 925–945. DOI: 10.1080/00918369.2021.1898803.

Makowski, A. C.; Mnich, E. E.; von-dem-Knesebeck, O. (2018): SSDS, Self-Stigma of Depression Scale - deutsche Fassung (PSYNDEX Tests Info), Self-Stigma of Depression Scale (SSDS; Barney, L. J., Griffiths, K. M., Christensen, H. & Jorm, A. F., 2010) - German version/author, Synonym(e): SSDS-D.

McGuire, Jenifer K.; Beek, Titia F.; Catalpa, Jory M.; Steensma, Thomas D.; psychology, Medical; Life, APH - Aging & Later et al. (2018): The Genderqueer Identity (GQI) Scale: Measurement and validation of four distinct subscales with trans and LGBQ clinical and community samples in two countries. In STARTPAGE=1;ENDPAGE=16;ISSN=1553-2739;TITLE=International Journal of Transgenderism. DOI: four.

McGuire, J. K.; Berg, D.; Catalpa, J. M.; Morrow, Q. J.; Fish, J. N.; Nic Rider, G. et al. (2020): Utrecht Gender Dysphoria Scale - Gender Spectrum (UGDS-GS): Construct validity among transgender, nonbinary, and LGBQ samples. In Int J Transgend Health 21 (2), pp. 194–208. DOI: 10.1080/26895269.2020.1723460.

McKay, Elizabeth Anne; Placencio-Castro, Matias; Harris, Allyssa; Kelly-Weeder, Susan; Fontenot, Holly B. (2023): Development and psychometric testing of a scale to measure Sexual Minority-Inclusivity in Sex Education (SMISES). In Sex Res Soc Policy 20 (4), pp. 1483–1497. DOI: 10.1007/s13178-023-00794-3.

Merced, K.; Ohayagha, C.; Watson, J. D.; Perrin, P. B. (2022): Clinical utility and psychometric properties of the Ableist Microaggressions Scale. In Rehabilitation Psychology 11, p. 11. DOI: Using.

Mildner, Carolyn Ann (2001): Sexual minority identity formation and internalized homophobia in lesbians: A validation study of a new instrument and related variables. In Dissertation Abstracts International: Section B: The Sciences and Engineering 62 (5-B), p. 2494. DOI: Health.

Miranda Ramos, Mozer de; Cerqueira-Santos, Elder (2019): Negative Attitudes Toward Effeminacy Scale (NATE): Adaptation and evidence of validity in Brazil. In Escala de Atitudes Negativas sobre Afeminacao (ANA): Adaptacao e evidencias de validade no Brasil 50 (2), No-Specified. DOI: Tests.

Moral de Rubia, José; Martínez Sulvarán, Juan Oswaldo (2012): Validación de la Escala de 10 ítems de Actitud hacia la Homosexualidad (EAH-10). In Revista de Psicología Social. DOI: Social.

Mozumder, M. K. (2017): Validation of Bengali perceived stress scale among LGBT population. In BMC Psychiatry 17 (1), p. 314. DOI: 10.1186/s12888-017-1482-0.

Mutchler, M. G.; Wagner, G. J.; McDavitt, B.; Woldetsadik, M. A.; Kegeles, S. M.; El-Khoury, C. et al. (2022): Psychometric Properties of a Scale on Judgmental Communication with Peers About Sex Among YMSM and Their Close Friends in Lebanon. In Aids Behav 26 (9), pp. 3089–3098. DOI: 10.1007/s10461-022-03640-6.

Nielsen, Sarah Elizabeth; Harbke, Colin R.; Herbstrith, Julie C. (2022): Uncovering bi-as: Developing new measures of binegativity. In Psychology of Sexual Orientation and Gender Diversity 9 (3), pp. 343–357. DOI: 10.1037/sgd0000399.

Nolet, K.; Guay, J. P.; Bergeron, S. (2021): Validation of the French-Canadian Version of the Sexual Inhibition and Sexual Excitation Scales-Short Form (SIS/SES-SF): Associations With Sexual Functioning, Sociosexual Orientation, and Sexual Compulsivity. In Sexual Medicine 9(4) (no pagination) (100374). DOI: 10.1016/j.esxm.2021.100374.

Ozturk, Pinar; Kindap, Yeliz (2011): Turkish adaptation of the Lesbian Internalized Homophobia Scale: A study of validity and reliability. In Lezbiyenlerde icsellectirilmis homofobi olceginin psikometrik ozelliklerinin incelenmesi 14 (28), pp. 24–35. DOI: Scales.

Peterson, Anne V. (1981): Emotional intimacy among college males: A construct validation study of the Personal Anxiety factor of the Homosexuality Attitude Scale. In Dissertation Abstracts International: Section B: The Sciences and Engineering 42 (1-B), p. 432. DOI: Scales.

Pineda Roa, Carlos Alejandro (2016): Psychometric properties of a test homonegativity internalized in Colombian's homosexual and bisexual men. In Propiedades psicometricas de una prueba de homonegatividad internalizada en varones homosexuales y bisexuales colombianos 33 (1), pp. 47–65. DOI: 10.14482/psdc.33.1.8074.

Ranjit, Yerina S.; Krishnan, Archana; Earnshaw, Valerie A.; Weikum, Damian; Ferro, Enrico G.; Sanchez, Jorge; Altice, Frederick L. (2023): Psychometric evaluation and validation of the HIV Stigma Scale in Spanish among men who have sex with men and transgender women. In Stigma and Health 8 (4), pp. 437–444. DOI: 10.1037/sah0000302.

Rendina, H. J.; Lopez-Matos, J.; Wang, K.; Pachankis, J. E.; Parsons, J. T. (2019): The Role of Self-Conscious Emotions in the Sexual Health of Gay and Bisexual Men: Psychometric Properties and Theoretical Validation of the Sexual Shame and Pride Scale. In Journal of sex research 56 (4-5), pp. 620–631. DOI: 10.1080/00224499.2018.1453042.

Rosser, B. Simon; Ross, Michael W. (1989): A Gay Life Events Scale (GALES) for homosexual men. In Journal of Gay & Lesbian Psychotherapy 1 (2), pp. 87–101. DOI: 10.1300/J236v01n02_07.

Rubia, José Moral dela; O, Adrián Valle dela; Gómez, Enrique Martínez (2013): Evaluation of rejection toward homosexuality in students of medicine and psychology based on three conceptually related scales. In Psicología desde el Caribe 30 (3). DOI: Norte.

Santos, Jean Jesus; Cerqueira-Santos, Elder (2023): Sexual orientation beliefs scale (sobs): Adaptation and validity evidence in the brazilian context. In Sex Res Soc Policy. DOI: 10.1007/s13178-023-00889-x.

Sarno, Elissa L.; Mohr, Jonathan J. (2016): Adapting the multigroup ethnic identity measure to assess LGB group identity. In Psychology of Sexual Orientation and Gender Diversity 3 (3), pp. 293–303. DOI: 10.1037/sgd0000173.

Sauceda, J. A.; Paul, J. P.; Gregorich, S. E.; Choi, K. H. (2016): Assessing Collectivism in Latino, Asian/Pacific Islander, and African American Men Who Have Sex With Men: A Psychometric Evaluation. In AIDS Educ Prev 28 (1), pp. 11–25. DOI: 10.1521/aeap.2016.28.1.11.

Schaefer, S. (1975): FSSP-L, FRAGEBOGEN ZU SEXUELLEN UND SOZIALEN PROBLEMEN VON LESBIERINNEN (PSYNDEX Tests Review), Psychosexual Problems Questionnaire for Lesbians/zpid. DOI: Tests.

Scheim, A. I.; Bauer, G. R. (2019): The Intersectional Discrimination Index: Development and validation of measures of self-reported enacted and anticipated discrimination for intercategorical analysis. In Soc Sci Med 226, pp. 225–235. DOI: 10.1016/j.socscimed.2018.12.016.

Schnarrs, P. W.; Dorri, A. A.; Yockey, R. A.; Stone, A. L.; Russell, S. T.; Rosenberger, J. G. (2023): The Sexual and Gender Minority Adverse Childhood Experiences Scale. In Am J Prev Med 65 (6), pp. 1050–1058. DOI: 10.1016/j.amepre.2023.08.006.

Schudson, Z. C.; van Anders, S. M.: Gender/sex diversity beliefs: Scale construction, validation, and links to prejudice. In Group Process Intergroup Relat. DOI: 10.1177/1368430220987595.

Seagal, Janel Dina (2001): Identity among members of stigmatized groups: A double-edged sword. In Dissertation Abstracts International: Section B: The Sciences and Engineering 62 (3-B), p. 1647. DOI: Social.

Sheffield, Alexis; Tung, Irene; Berona, Johnny; Northrup, Jessie B.; Nannini, Sierra; Hipwell, Alison E.; Keenan, Kate (2022): Factor structure of the outness inventory in a sample of black and white lesbian and bisexual young adult women. In Journal of Gay & Lesbian Mental Health (Pagination). DOI: 10.1080/19359705.2022.2046973.

Skidmore, S. J.; Lefevor, G. T.; Huynh, K. D.; Berg, C. O. (2024): Development and Initial Validation of Scales for Coming Out Vigilance and Positive Coming Out Responses. In Sexuality & Culture 28, pp. 771–793. DOI: 10.1007/s12119-023-10144-5.

Song, C.; Buysse, A.; Zhang, W. H.; Lu, C.; Zhao, M.; Dewaele, A. (2022): The Factor Structure and Measurement Invariance of the Coping Inventory for Stressful Situations in a Sexual Minority Population. In J Homosex, pp. 1–16. DOI: 10.1080/00918369.2022.2122366.

Song, C.; Buysse, A.; Zhang, W. H.; Lu, C.; Zhao, M.; Dewaele, A. (2024): The Factor Structure and Measurement Invariance of the Coping Inventory for Stressful Situations in a Sexual Minority Population. In J Homosex 71 (2), pp. 512–527. DOI: 10.1080/00918369.2022.2122366.

Tebbe, E. A.; Moradi, B.; Wilson, E.; Bell, H. L.; Connelly, K.; Lenzen, A. (2021): Development and psychometric evaluation of the Sexual Minority Women's Sexual Objectification Experiences Scale. In J Couns Psychol 25, p. 25. DOI: 10.1037/cou0000547.

Totenhagen, Casey J.; Randall, Ashley K.; Leon, Gabriel A.; Carroll, Mackenzie (2023): Dyadic Coping Inventory-Sexual Minority Stress: A scale validation with lesbian, gay, and bi+ men and women in same- and different-gender couples. In Psychology of Sexual Orientation and Gender Diversity, No Pagination Specified. DOI: 10.1037/sgd0000643.

Ubeda-Colomer, J.; Gil-Quintana, J.; Pereira-Garcia, S.; Lopez-Canada, E.; Perez-Samaniego, V.; Devis-Devis, J. (2020): Development and validation of the Barriers to Physical Activity and Sport Questionnaire for lesbian, gay, bisexual, transgender and queer/questioning persons. In Public Health 185, pp. 202–208. DOI: 10.1016/j.puhe.2020.05.020.

Wade, Ryan M. (2018): Racialized sexual discrimination (RSD) and psychological wellbeing among young Black gay/bisexual men (YBGM). In Dissertation Abstracts International: Section B: The Sciences and Engineering 79 (12-B(E)), No-Specified. DOI: Health.

Wiseman, Marcie C. (2010): Development and validation of the Sexual Minority Men's Body Objectification Experiences Scale. In Dissertation Abstracts International: Section B: The Sciences and Engineering 71 (3-B), p. 2073. DOI: 2200.

Zsofia, Torok; Marton, Rakovics; Melinda, Penzes; Andras, Terebessy; Vince, Pongor; Peter, Csepe (2018): Hungarian adaptation of the Modern Homonegativity Scale. In A Modern Homonegativitas Skala magyarorszagi adaptacioja 19 (4), pp. 357–383. DOI: 10.1556/0406.19.2018.4.009.

## A4

None.

## A5

Baiocco, Roberto; Salvati, Marco; Carone, Nicola; Ioverno, Salvatore; Nappa, Maria Rosaria; Pistella, Jessica (2018): Positive identity in sexual minorities: A contribution to the Italian validation of the Lesbian, Gay, and Bisexual Positive Identity Measure (LGB-PIM). In Identita positiva in persone lesbiche, gay e bisessuali: Un contributo alla validazione italiana della Lesbian, Gay, and Bisexual Positive Identity Measure (LGB-PIM) 45 (4), pp. 953–976. DOI: Tests.

Bulboaca, Gabriel; Robu, Viorel (2023): Homosexuality Attitude Scale (HAS): Psychometric properties of the Romanian version. In Revista de Psihologie 69 (1), pp. 5–39. DOI: Study.

Gomez, Fabiola; Cumsille, Patricio; Gonzalez, Roberto (2022): Validation of the short-version of the Modern Homonegativity Scale in Chilean youth. In Psykhe: Revista de la Escuela de Psicologia 31 (2), pp. 1–16. DOI: 10.7764/psykhe.2020.22563.

Gomez, Fabiola; Guzman-Gonzalez, Monica; Barrientos, Jaime; Frost, David; Espinoza-Tapia, Ricardo; Rojas, Lusmenia Garrido (2023): Psychometric properties of a Spanish version of the Revised Internalized Homonegativity Scale in gay men and lesbian women. In Revista Iberoamericana de Diagnostico y Evaluacion Psicologica 67 (1), pp. 35–46. DOI: 10.21865/RIDEP67.1.03.

Pineda Roa, Carlos Alejandro (2016): Psychometric properties of a test homonegativity internalized in Colombian's homosexual and bisexual men. In Propiedades psicometricas de una prueba de homonegatividad internalizada en varones homosexuales y bisexuales colombianos 33 (1), pp. 47–65. DOI: 10.14482/psdc.33.1.8074.

## A6

Algarin, Angel B.; Smith, Laramie R.; Pines, Heather A.; Zapien-Vasquez, Monica F.; Padilla-Garcia, Rosario; Navarro-Alvarez, Samuel; Pitpitan, Eileen V. (2022): The Gender Minority Stress and Resilience Measure: Psychometric Validity for Use in Spanish. In Transgender Health. DOI: 10.1089/trgh.2021.0190.

Branstrom, R.; Pachankis, J. (2018): Validating the Syndemic Threat Surrounding Sexual Minority Men's Health in a Population-Based Study. In Ann. Behav. Med. 52, S220-S220. DOI: Threat.

Dickey-Chasins, Harriett B. (2001): The development of the Shame Due to Heterosexism Scale. In Dissertation Abstracts International: Section B: The Sciences and Engineering 61 (9-B), p. 5048. DOI: 2200.

Floyd, Rebecca M. (2014): Defining the minority stress scale of the cultural assessment for risk of suicide, for use with multiple minority groups. In Dissertation Abstracts International: Section B: The Sciences and Engineering 74 (11-B(E)), No-Specified. DOI: Health.

Oshi-Ojuri, Morenike F. (2015): A quantitative look at the impact of microaggressions on the intersecting identities of African American lesbians. In Dissertation Abstracts International: Section B: The Sciences and Engineering 76 (4-B(E)), No-Specified. DOI: Health.

Peterson, Trica Luanne (2006): Understanding the connection between minority stress and identity development in self-identified and questioning lesbians. In Dissertation Abstracts International: Section B: The Sciences and Engineering 66 (8-B), p. 4496. DOI: Health.

Proescholdbell, Rae Jean E. (2003): Psychological sense of community: Development of measures of its theoretical components. In Dissertation Abstracts International: Section B: The Sciences and Engineering 64 (3-B), p. 1504. DOI: Health.

Resnick, Courtney A. (2018): Measuring experiences of LGBT microaggressions in the workplace: Initial development and validation of the microaggression experiences at work scale. In Dissertation Abstracts International Section A: Humanities and Social Sciences 78 (8-A(E)), No-Specified. DOI: 3600.

Reuben, Jacqueline Rose (2023): Intersectional stigma, self-efficacy, depression, and resilience: A Rasch analysis.DP - 2023. In Dissertation Abstracts International: Section B: The Sciences and Engineering 84 (8-B), No Pagination Specified. DOI: Study.

Rivera, Z.; Simon, V. A.; Partridge, T. (2022): Psychometric Properties of the Sexual Subjectivity Inventory among Lesbian, Gay, and Bisexual Emerging Adults. In Journal of sex research, pp. 1–11. DOI: 10.1080/00224499.2022.2091100.

Rivera, Z.; Simon, V. A.; Partridge, T. (2023): Psychometric Properties of the Sexual Subjectivity Inventory among Lesbian, Gay, and Bisexual Emerging Adults. In Journal of sex research 60 (1), pp. 126–136. DOI: 10.1080/00224499.2022.2091100.

Smith, Mark Spencer (2007): The development and psychometric evaluation of a rapid assessment instrument measuring hardiness in lesbian, gay, bisexual, and transgender persons. In Dissertation Abstracts International Section A: Humanities and Social Sciences 67 (10-A), p. 3983. DOI: Health.

Sultana, A.; Farooq, R. (2024): Towards the measurement of stereotype threat: scale development and validation. In - Glob. Knowl. Mem. Commun.

Tran, Ha Thi Ngoc (2016): Internalized Homonegativity: The psychometric structure of the short scale and HIV/SIT associated behaviors. In Dissertation Abstracts International: Section B: The Sciences and Engineering 77 (2-B(E)), No-Specified. DOI: Health.

Truong, Nhan (2010): The role of masculinity-based stress and minority stress in the mental health of Asian and Pacific Islander gay and bisexual men in the U.S. In Dissertation Abstracts International: Section B: The Sciences and Engineering 71 (3-B), p. 2096. DOI: Social.

Valle de la O., Adrian; Moral de la Rubia, Jose (2018): Construct and criterion validity of the 16-item scale for assessing internalized homonegativity. In Research on Hispanic psychology., Vol. 1, pp. 97–146. DOI: Tests.

Walch, Susan E.; Kennedy, Jada S.; Smith, Victoria A.; Smith, J.; Arena, Lanae M.; Hamilton, Michelle K.; Berger-Smith, Alexandra A. (2024): Psychological measurement in transgender and gender diverse populations: A comprehensive review of published self-report measures. In Journal of Projective Psychology & Mental Health 31 (1), pp. 35–46. DOI: Review.

Wei, Tao (2018): Measuring minority stigma among Chinese women with same-sex attraction. In Dissertation Abstracts International: Section B: The Sciences and Engineering 79 (7-B(E)), No-Specified. DOI: Health.

Wratten, S.; Finbow, C.; Hatton, S.; Jones, A. M.; Wells, J.; Young, A. (2022): EVERYONE SHOULD BE INVITED TO THE PROM: DIVERSIFYING PATIENT REPORTED OUTCOME MEASURES FOR LGBTQ plus INCLUSION. In Value in Health 25 (1), S236-S236.

Yadlosky, Lauren B. (2018): Assessing intersectional identity: Investigating the Scales of Contextualized Identity and Perceived Marginalization. In Dissertation Abstracts International: Section B: The Sciences and Engineering 79 (11-B(E)), No-Specified. DOI: 2100.

Zha, P.; Qureshi, R.; Porter, S.; Lin, H. (2022): Psychometric Evaluation of the Patient-Nurse Trust Scale among Sexual and Gender Minorities. In J Community Health Nurs 39 (4), pp. 238–250. DOI: 10.1080/07370016.2022.2080500.

## A7

Barros, Ana Carolina de; Morrison, Todd G. (2023): Development and validation of the Positive Bisexual Identity (PBI) scale. In Psychology & Sexuality 14 (1), pp. 73–93. DOI: 10.1080/19419899.2022.2057868.

Braitman, A. L.; Romano, K. A.; Heron, K. E.; Ehlke, S. J.; Shappie, A. T.; Lewis, R. J. (2021): Psychometric analysis and validity of the Daily Sexual Minority Stressors Scale among young adult same-sex female couples. In Psychological assessment 33 (11), pp. 1025–1037. DOI: 10.1037/pas0001073.

Camp, J.; Vitoratou, S.; Rimes, K. A. (2022): The Self-Acceptance of Sexuality Inventory (SASI): Development and Validation. In Psychology of Sexual Orientation and Gender Diversity 9 (1), pp. 92–109. DOI: 10.1037/sgd0000445.

Camp, Jake; Vitoratou, Silia; Rimes, Katharine A. (2022): The Self-Acceptance of Sexuality Inventory (SASI): Development and validation. In Psychology of Sexual Orientation and Gender Diversity 9 (1), pp. 92–109. DOI: 10.1037/sgd0000445.

Flebus, Giovanni Battista; Montano, Antonella; Barlascini, Luca (2011): The Multifactor Internalized Homophobia Inventory: Concurrent validation and sociodemographic features of a tool for the measuring of internalized homophobia. In Multifactor Internalized Homophobia Inventory: validazione concorrente e caratteristiche sociodemografiche di uno strumento per la misura dell'omofobia interiorizzata 17 (3), pp. 319–343. DOI: Scales.

Griffin, J. A.; Eldridge-Smith, E. D.; Kridel, M. M.; Draheim, A. A.; Stepleman, L. M. (2022): Validation of the Brief Sense of Community Scale in a lesbian, gay, and bisexual sample. In J. Community Psychol. 50(1), pp. 576–591. DOI: 10.1002/jcop.22638.

Hsieh, M. T.; Chen, J. S.; Lin, C. Y.; Yen, C. F.; Griffiths, M. D.; Huang, Y. T. (2021): Measurement Invariance of the Sexual Orientation Microaggression Inventory across LGB Males and Females in Taiwan: Bifactor Structure Fits the Best. In International Journal of Environmental Research & Public Health [Electronic Resource] 18 (20), p. 12. DOI: Using.

Maiolatesi, A. J.; Satyanarayana, S.; Branstrom, R.; Pachankis, J. E. (2023): Development and Validation of Two Abbreviated Intraminority Gay Community Stress Scales. In Assessment 30 (1), pp. 84–101. DOI: 10.1177/10731911211042933.

Maiolatesi, A. J.; Satyanarayana, S.; Branstrom, R.; Pachankis, J. E. (2023): Development and Validation of Two Abbreviated Intraminority Gay Community Stress Scales. In Assessment 30 (1), pp. 84–101. DOI: 10.1177/10731911211042933.

Torres, T. S.; Luz, P. M.; Bezerra, D. R. B.; Almeida-Brasil, C. C.; Marins, L. M. S.; Veloso, V. G. et al. (2021): Translation and validation in Brazilian Portuguese of the reactions to homosexuality scale. In Psychology, health & medicine 26(Supplement1), pp. 20–36. DOI: 10.1080/13548506.2021.1936580.

Zelaya, David G.; DeBlaere, Cirleen; Velez, Brandon L. (2023): Psychometric validation and extension of the LGBT people of Color Microaggressions Scale with a sample of sexual minority BIPOC college students. In Psychology of Sexual Orientation and Gender Diversity 10 (3), pp. 405–412. DOI: 10.1037/sgd0000553.

# Selection of Minority Stress Measurement Instruments: Excluded References by Exclusion Category

## Exclusion Categories:

E1: The instrument was not available in English or German.

E2: The construct did not address multiple dimensions of minority stress.

E3: The target population did not include at least both lesbian and gay adults.

E4: The target population focused on a specific subgroup.

## E1

Iniewicz, G., Sałapa, K., Wrona, M., & Marek, N. (2017). Minority stress among homosexual and bisexual individuals – from theoretical concepts to research tools: The Sexual Minority Stress Scale. Archives of Psychiatry and Psychotherapy, 19(3), 69–80. https://doi.org/10.12740/APP/75483

Kemer, G., Demirtaş, E. T., Pope, A. L., & Ummak, E. (2017). Psychometric Properties of the Lesbian, Gay, and Bisexual Identity Scale-Turkish (LGBIS-TR). Journal of Homosexuality, 64(12), 1632–1649. https://doi.org/10.1080/00918369.2016.1249741

Lingiardi, V., Baiocco, R., & Nardelli, N. (2012). Measure of internalized sexual stigma for lesbians and gay men: A new scale. Journal of Homosexuality, 59(8), 1191–1210. https://doi.org/10.1080/00918369.2012.712850

Mijas, M., & Koziara, K. (2020). Polska adaptacja Kwestionariusza Codziennych Doświadczeń Heteroseksizmu [Polish adaptation of the Daily Heterosexist Experiences Questionnaire]. Psychiatria Polska, 54(1), 137–152. https://doi.org/10.12740/PP/97215

Morell-Mengual, V., Gil-Llario, M. D., Ballester-Arnal, R., & Salmerón-Sanchéz, P. (2017). Spanish Adaptation and Validation of the Short Internalized Homonegativity Scale (SIHS). Journal of Sex & Marital Therapy, 43(4), 298–305. https://doi.org/10.1080/0092623X.2016.1149128

Oliveira, J. M. de, Lopes, D., Costa, C. G., & Nogueira, C. (2012). Lesbian, Gay, and Bisexual Identity Scale (LGBIS): Construct validation, sensitivity analyses and other psychometric properties. The Spanish Journal of Psychology, 15(1), 334–347. https://doi.org/10.5209/rev_SJOP.2012.v15.n1.37340

Pitonak, M., & Cihak, M. (2023). Understanding broader LGB+ identity from a post-socialist perspective: Assessment of validity of the lesbian, Gay, and Bisexual Identity Scale (LGBIS) on a Czech sample. Ceskoslovenska Psychologie: Casopis Pro Psychologickou Teorii a Praxi, 67(2), 121–140. https://doi.org/10.51561/cspsych.67.2.121

Ronzon-Tirado, R., Charak, R., & Cano-Gonzalez, I. (2023). Daily Heterosexist Experiences in LGBTQ+ Adults from Spain: Measurement, Prevalence, and Clinical Implications. Intervencion Psicosocial, 32(1), 1–10. https://doi.org/10.5093/pi2022a15

Yen, C.‑F., Huang, Y.‑T., Potenza, M. N., Tsai, T.‑T., Lin, C.‑Y., & Tsang, H. W. H. (2021). Measure of Internalized Sexual Stigma for Lesbians and Gay Men (MISS-LG) in Taiwan: Psychometric Evidence from Rasch and Confirmatory Factor Analysis. International Journal of Environmental Research and Public Health, 18(24). https://doi.org/10.3390/ijerph182413352

## E2

Barburoğlu, Y., Çürükvelioğlu‐Köksal, E., Özgülük Üçok, S. B., Gandhi, Y., Lannutti, P. J., & Randall, A. K. (2024). Measuring positive LGBTQ + identity: Psychometric properties of the Turkish Lesbian, Gay, and Bisexual Positive Identity Measure. Personal Relationships, 31(3), 628–647. https://doi.org/10.1111/pere.12561

Bauerband, L. A [Loren A.], Teti, M., & Velicer, W. F. (2019). Measuring minority stress: invariance of a discrimination and vigilance scale across transgender and cisgender LGBQ individuals. Psychology & Sexuality, 10(1), 17–30. https://doi.org/10.1080/19419899.2018.1520143

Camp, J., Vitoratou, S., & Rimes, K. A. (2022). The Self-Acceptance of Sexuality Inventory (SASI): Development and validation. Psychology of Sexual Orientation and Gender Diversity, 9(1), 92–109. https://doi.org/10.1037/sgd0000445

Crowell, K. A. (2014). The mental health disparity among nonheterosexuals: Risk, resiliency, and new perspectives to consider in the context of mormonism. https://digitalcommons.usu.edu/etd/2127

Day, N., Meglich, P., & Porter, T. H. (2022). Measuring Bullying in Sexual Minorities: Testing Two Bullying Scales in an LGB Sample. Journal of Homosexuality, 69(7), 1160–1184. https://doi.org/10.1080/00918369.2021.1909393

Di Luigi, G., Claréus, B., Mejias Nihlén, T., Malmquist, A., Wurm, M., & Lundberg, T. (2024). Psychometric Exploration of the Swedish Translation of the Sexual Orientation Microaggressions Scale (SOMS), and a Commentary on the Validity of the Construct of Microaggressions. Journal of Homosexuality, 71(14), 3230–3253. https://doi.org/10.1080/00918369.2023.2284809

Fox, C., & Asquith, N. L. (2018). Measuring the Tangible Fear of Heterosexist Violence. Journal of Interpersonal Violence, 33(6), 980–1007. https://doi.org/10.1177/0886260515614279

Gallo-Barrera, Y. D., & Campo-Arias, A. (2022). Sexual Orientation Experiences of Discrimination: Dimensionality, internal consistency, and nomological validity in a diverse Colombian population. https://doi.org/10.1101/2022.02.06.22269563

Galupo, M. P., & Bauerband, L. A [L. A.] (2016). Sexual Orientation Reflection and Rumination Scale: Development and psychometric evaluation. Stigma and Health, 1(1), 44–58. https://doi.org/10.1037/sah0000017

Griffin, J. A., Eldridge-Smith, E. D., Kridel, M. M., Draheim, A. A., & Stepleman, L. M. (2022). Validation of the Brief Sense of Community Scale in a lesbian, gay, and bisexual sample. Journal of Community Psychology, 50(1), 576–591. https://doi.org/10.1002/jcop.22638

Herek, G. M. Cogan, J. C. Gillis, J. R. Glunt, E. K. (1998). Correlates of Internalized Homophobia in a Community Sample of Lesbians and Gay Men. Journal of the Gay & Lesbian Medical Assn(2), 17–25.

Herek, G. M., Gillis, J. R., & Cogan, J. C. (2009). Internalized stigma among sexual minority adults: Insights from a social psychological perspective. Journal of Counseling Psychology, 56(1), 32–43. https://doi.org/10.1037/a0014672

Hsieh, M.‑T., Chen, J.‑S., Lin, C.‑Y., Yen, C.‑F., Griffiths, M. D., & Huang, Y.‑T. (2021). Measurement Invariance of the Sexual Orientation Microaggression Inventory across LGB Males and Females in Taiwan: Bifactor Structure Fits the Best. International Journal of Environmental Research and Public Health, 18(20). https://doi.org/10.3390/ijerph182010668

Jackson, S. D., & Mohr, J. J. (2016). Conceptualizing the closet: Differentiating stigma concealment and nondisclosure processes. Psychology of Sexual Orientation and Gender Diversity, 3(1), 80–92. https://doi.org/10.1037/sgd0000147

Keum, B. T., Maurin-Waters, E., & Chong, E. S. K. (2023). The Mental Health Costs of Online Heterosexism Among LGBTQ+ Individuals: Development and Initial Validation of the Perceived Online Heterosexism Scale. Archives of Sexual Behavior, 09, 525–541. https://doi.org/10.1007/s10508-023-02733-0

Kler, S., Arora, S., & Le, T. P. (2023). Validation of the Multidimensional Scale of Perceived Social Support (MSPSS) Among LGBTQ+ People of Color. Measurement and Evaluation in Counseling and Development, 56(4), 283–296. https://doi.org/10.1080/07481756.2022.2109174

Lasser, J., Ryser, G. R., & Price, L. R. (2010). Development of a lesbian, gay, bisexual visibility management scale. Journal of Homosexuality, 57(3), 415–428. https://doi.org/10.1080/00918360903543154

Meidlinger, P. C., & Hope, D. A. (2014). Differentiating disclosure and concealment in measurement of outness for sexual minorities: The Nebraska Outness Scale. https://digitalcommons.unl.edu/cgi/viewcontent.cgi?article=1875&context=psychfacpub

Murray, J. C., & Dailey, S. F. (2020). Development of the LGBTQ Belongingness Attainment Scale. Journal of LGBT Issues in Counseling, 14(2), 100–117. https://doi.org/10.1080/15538605.2020.1753622

Nadal, K. L. (2019). Measuring LGBTQ Microaggressions: The Sexual Orientation Microaggressions Scale (SOMS) and the Gender Identity Microaggressions Scale (GIMS). Journal of Homosexuality, 66(10), 1404–1414. https://doi.org/10.1080/00918369.2018.1542206

Parmenter, J. G., & Galliher, R. V. (2024). Development and initial validation of the LGBTQ+ Community Resilience and Inequity Scale. Psychology of Sexual Orientation and Gender Diversity, 11(1), 56–68. https://doi.org/10.1037/sgd0000601

Pinel, E. C. (1999). Stigma consciousness: The psychological legacy of social stereotypes. Journal of Personality and Social Psychology, 76(1), 114–128. https://doi.org/10.1037//0022-3514.76.1.114

Riggle, E. D. B., Folberg, A. M., Richardson, M. T., & Rostosky, S. S. (2023). A measure of hypervigilance in LGBTQ-identified individuals. Stigma and Health, 8(4), 476–486. https://doi.org/10.1037/sah0000306

Riggle, E. D. B., Mohr, J. J., Rostosky, S. S., Fingerhut, A. W., & Balsam, K. F. (2014). A multifactor Lesbian, Gay, and Bisexual Positive Identity Measure (LGB-PIM). Psychology of Sexual Orientation and Gender Diversity, 1(4), 398–411. https://doi.org/10.1037/sgd0000057

Seabra, D., Gato, J., Petrocchi, N., Carreiras, D., & do Céu Salvador, M. (2024). Stigma Consciousness Questionnaire (SCQ-PT): Validation in a Portuguese Sample of Sexual Minorities. Sexuality Research and Social Policy, 21(1), 148–160. https://doi.org/10.1007/s13178-023-00859-3

Smith, M. S., & Gray, S. W. (2009). The Courage to Challenge: A New Measure of Hardiness in LGBT Adults. Journal of Gay & Lesbian Social Services, 21(1), 73–89. https://doi.org/10.1080/10538720802494776

Skidmore, S. J., Lefevor, G. T., Huynh, K. D., & Berg, C. O. (2024). Development and Initial Validation of Scales for Coming Out Vigilance and Positive Coming Out Responses. Sexuality & Culture, 28, 771–793. https://doi.org/10.1007/s12119-023-10144-5

Strizzi, J., Fernández-Agis, I., Parrón-Carreño, T., & Alarcón-Rodríguez, R. (2016). Enacted Sexual Stigma, Stigma Consciousness, and Subjective Happiness Scale Adaptation: A Two-Country Study. Journal of Interpersonal Violence, 31(2), 316–338. https://doi.org/10.1177/0886260514555372

Sullivan, T. J., Huang, H., Vilkin, E., Brandt, S. A., & Davila, J. (2023). A measurement invariance/equivalence study of sexual minority stress measures across sexual orientation, gender identity, and race/ethnicity. Psychology of Sexual Orientation and Gender Diversity, Advance online publication. https://doi.org/10.1037/sgd0000681

Swann, G., Bettin, E., Ryan, D. T., Clifford, A., Newcomb, M. E., Whitton, S. W., & Mustanski, B. (2022). The Sexual Orientation Microaggression Inventory Short Form (SOMI-SF): Validation in Three Samples of Racially/Ethnically Diverse Sexual Minority Youth. Sexuality Research and Social Policy. Advance online publication. https://doi.org/10.1007/s13178-022-00695-x

Toscano, M. E. (2022). Reconceptualization and Validation of a Measure of Self-Acceptance Within the LGBTQ + Communities. Journal of LGBTQ Issues in Counseling, 16(3), 285–299. https://doi.org/10.1080/26924951.2022.2057389

Toscano, M. E. (2024). The Continued Exploration of Self-Acceptance: Creating a Normative Sample and Examining the Incremental Validity of the FSA-SGI. Journal of LGBTQ Issues in Counseling, 18(1), 39–57. https://doi.org/10.1080/26924951.2023.2293294

Vidales, C. A., Fernando, G. A., & Vogel, D. L. (2023). Perceived Familial Stigma of Sexuality. Measurement and Evaluation in Counseling and Development, 56(1), 33–47. https://doi.org/10.1080/07481756.2022.2030238

Wegner, R., & Wright, A. J. (2016). A psychometric evaluation of the homonegative microaggressions scale. Journal of Gay & Lesbian Mental Health, 20(4), 299–318. https://doi.org/10.1080/19359705.2016.1177627

Wickham, R. E., Gutierrez, R., Giordano, B. L., Rostosky, S. S., & Riggle, E. D. B. (2021). Gender and Generational Differences in the Internalized Homophobia Questionnaire: An Alignment IRT Analysis. Assessment, 28(4), 1159–1172. https://doi.org/10.1177/1073191119893010

Wright, A. J., & Wegner, R. T. (2012). Homonegative Microaggressions and Their Impact on LGB Individuals: A Measure Validity Study. Journal of LGBT Issues in Counseling, 6(1), 34–54. https://doi.org/10.1080/15538605.2012.648578

## E3

Broshek, C. E., Kurtz, A. J., Udupa, N. S., Chang, C. J., Rogers, M. L., Feinstein, B. A., Selby, E. A., Gratz, K. L., Tull, M. T., Joiner, T. E., & Jeon, M. E. (2024). Psychometric Evaluation of the Heterosexist Harassment, Rejection, and Discrimination Scale: Identification of a Unidimensional 11-Item Scale. Assessment, 10731911241293222. https://doi.org/10.1177/10731911241293222

Brown, C. A., Sullivan, P. S., Stephenson, R., Baral, S. D., Bekker, L.‑G., Phaswana-Mafuya, N. R., Simbayi, L. C., Sanchez, T., Valencia, R. K., Zahn, R. J., & Siegler, A. J. (2021). Developing and validating the Multidimensional Sexual Identity Stigma Scale among men who have sex with men in South Africa. Stigma and Health, 6(3), 277–286. https://doi.org/10.1037/sah0000294

Choi, A. Y., Merrill, C. R. S., & Israel, T [Tania] (2017). Factor structure of the Internalized Homonegativity Inventory (IHNI). Psychology of Sexual Orientation and Gender Diversity, 4(4), 491–498. https://doi.org/10.1037/sgd0000245

Cohen, M. A. (2014). Psychometric Properties of the Nungesser Homosexual Attitudes Inventory and its Relation to Health Risks Among Gay Men. Temple University. http://hdl.handle.net/20.500.12613/2706 https://doi.org/10.34944/DSPACE/2688

Currie, M. R., Cunningham, E. G., & Findlay, B. M. (2004). The Short Internalized Homonegativity Scale: Examination of the Factorial Structure of a New Measure of Internalized Homophobia. Educational and Psychological Measurement, 64(6), 1053–1067. https://doi.org/10.1177/0013164404264845

Mayfield, W. (2001). The development of an Internalized Homonegativity Inventory for gay men. Journal of Homosexuality, 41(2), 53–76. https://doi.org/10.1300/J082v41n02_04

Nungesser, L. G. (1983). Homosexual acts, actors, and identities. Praeger.

Ogunbajo, A., Iwuagwu, S., Williams, R., Biello, K. B., Kahler, C. W., Sandfort, T. G. M., & Mimiaga, M. J. (2020). Validation of depressive symptoms, social support, and minority stress scales among gay, bisexual, and other men who have with men (GBMSM) in Nigeria, Africa: A mixed methods approach. BMC Public Health, 20(1), 1023. https://doi.org/10.1186/s12889-020-09127-0

Provence, M. M., Parent, M. C., Rochlen, A. B., & Chester, M. R. (2019). Development of the Gay Male Heterophobia Scale. Psychology of Men & Masculinity, 20(1), 44–54. https://doi.org/10.1037/men0000158

Ross, M. W [M. W.], & Rosser, B. R. (1996). Measurement and correlates of internalized homophobia: A factor analytic study. Journal of Clinical Psychology, 52(1), 15–21. https://doi.org/10.1002/(SICI)1097-4679(199601)52:1<15::AID-JCLP2>3.0.CO;2-V

Shidlo, A. Internalized Homophobia: Conceptual and Empirical Issues in Measurement. In Lesbian and Gay Psychology: Theory, Research, and Clinical Applications (pp. 176–205). https://doi.org/10.4135/9781483326757.n10

Smallwood, S. W., Spencer, S. M., Ingram, L. A., Thrasher, J. F., & Thompson-Robinson, M. V. (2017). Different Dimensions: Internalized Homonegativity Among African American Men Who Have Sex With Men in the Deep South. Journal of Homosexuality, 64(1), 45–60. https://doi.org/10.1080/00918369.2016.1172869

Smolenski, D. J., Diamond, P. M., Ross, M. W [Michael W.], & Rosser, B. R. S. (2010). Revision, criterion validity, and multigroup assessment of the reactions to homosexuality scale. Journal of Personality Assessment, 92(6), 568–576. https://doi.org/10.1080/00223891.2010.513300

Szymanski, D. M [Dawn M.] (2006). Does Internalized Heterosexism Moderate the Link Between Heterosexist Events and Lesbians' Psychological Distress? Sex Roles, 54(3-4), 227–234. https://doi.org/10.1007/s11199-006-9340-4

Szymanski, D. M [D. M.], & Chung, Y. B. (2001). The Lesbian Internalized Homophobia Scale: A rational/theoretical approach. Journal of Homosexuality, 41(2), 37–52. https://doi.org/10.1300/J082v41n02_03

Theodore, J. L. (2004). Measurements and characteristics of internalized homophobia among gay men. Ferkauf Graduate School of Psychology. https://hdl.handle.net/20.500.12202/772

Theodore, J. L., Shidlo, A., Zemon, V., Foley, F. W., Dorfman, D., Dahlman, K. L., & Hamid, S. (2013). Psychometrics of an internalized homophobia instrument for men. Journal of Homosexuality, 60(4), 558–574. https://doi.org/10.1080/00918369.2013.760304

Tran, H., Ross, M. W [Michael W.], Diamond, P. M., Berg, R. C., Weatherburn, P., & Schmidt, A. J. (2018). Structural Validation and Multiple Group Assessment of the Short Internalized Homonegativity Scale in Homosexual and Bisexual Men in 38 European Countries: Results From the European MSM Internet Survey. Journal of Sex Research, 55(4-5), 617–629. https://doi.org/10.1080/00224499.2017.1380158

Weibley, S. M., & Hindin, M. (2013). Self-Identified Lesbian Internalized Homophobia Scale. In T. D. Fisher, C. M. Davis, & W. L. Yarber (Eds.), Handbook of Sexuality-Related Measures (pp. 400–402). Routledge.

## E4

Cramer, R. J., Kaniuka, A. R., Lange, T. M., Brooks, B. D., Feinstein, B. A., & Hilgeman, M. M. (2022). A pilot evaluation of sexual and gender minority identity measures in a treatment-engaged military veteran sample. American Journal of Orthopsychiatry, 92(4), 442–451. https://doi.org/10.1037/ort0000627

Feinstein, B. A., Khan, A., Chang, C. J., & Miller, S. A. (2023). Use of the Heterosexist Harassment, Rejection, and Discrimination Scale With Different Sexual Orientation, Gender, and Racial/Ethnic Groups: An Examination of Measurement Invariance. Assessment, 30(8), 2605–2615. https://doi.org/10.1177/10731911231156135

Ramos, J. M. (2024). Development of the Emerging Adult Inventory of Sexuality and Gender Minority Stress.Dp - 2024. Dissertation Abstracts International Section a: Humanities and Social Sciences, 85(3-A). https://surface.syr.edu/context/etd/article/2740/type/native/viewcontent

Smith, E. R., Perrin, P. B., & Sutter, M. E. (2020). Factor analysis of the heterosexist harassment, rejection, and discrimination scale in lesbian, gay, bisexual, transgender, and queer people of colour. International Journal of Psychology : Journal International De Psychologie, 55(3), 405–412. https://doi.org/10.1002/ijop.12585

## E1E2

Assink, M., & Bos, H. M. W. (2024). Gay Community Stress in Sexual Minority Men and Women: A Validation Study in the Netherlands. Journal of Homosexuality, 71(9), 2256–2285. https://doi.org/10.1080/00918369.2023.2231119

Gençöz, T., & Yüksel, M. (2006). Psychometric properties of the Turkish version of the internalized homophobia scale. Archives of Sexual Behavior, 35(5), 597–602. https://doi.org/10.1007/s10508-006-9063-1

Kittiteerasack, P., Matthews, A. K., & Park, C. (2024). Psychometric properties of the Thai version of the Internalized sexual stigma scale for research on lesbian, gay, bisexual, and transgender (LGBT) populations. Psychology & Sexuality, 15(3), 305–317. https://doi.org/10.1080/19419899.2021.2000013

Mak, W. W. S., & Cheung, R. Y. M. (2010). Self-stigma among concealable minorities in Hong Kong: Conceptualization and unified measurement. The American Journal of Orthopsychiatry, 80(2), 267–281. https://doi.org/10.1111/j.1939-0025.2010.01030.x

Seabra, D., Gato, J., Petrocchi, N., Carreiras, D., Azevedo, J., Martins, L., & Salvador, M. d. C. (2024). Everyday Discrimination Scale: Dimensionality in a Portuguese community sample and specific versions for sexual and gender minority. Current Psychology, 43(12), 10850–10861. https://doi.org/10.1007/s12144-023-05195-8

Silva, W. A. D., Do Bú, E. A., Brito, T. R. d. S., Figueiredo, C. V. de, & Pereira, C. R. (2025). The impact of sexual orientation microaggressions on the mental health of Brazilian lesbian, gay, and bisexual individuals. Psychology of Sexual Orientation and Gender Diversity. Advance online publication. https://doi.org/10.1037/sgd0000807

Xu, W., Zheng, L., Xu, Y., & Zheng, Y. (2017). Internalized homophobia, mental health, sexual behaviors, and outness of gay/bisexual men from Southwest China. International Journal for Equity in Health, 16(1), 36. https://doi.org/10.1186/s12939-017-0530-1

Yodlorchai, R., Suttiwan, P., Walch, S., & Ngamake, S. (2024). Developing and Validating Thai Sexual Orientation Microaggression and Microaffirmation Scales. Journal of Homosexuality, 71(13), 3023–3051. https://doi.org/10.1080/00918369.2023.2275301

## E1E3

Flebus, G. B., & Montano, A. (2009). Contribution to the Italian translation and adaptation of the Lesbian Internalized Homophobia Scale of Szymanski and Chung. Bollettino Di Psicologia Applicata(258), 23–32

Horne, S. G [Sharon G.], Maroney, M. R., Geiss, M. L., & Dunnavant, B. R. (2017). The reliability and validity of a Russian version of the Lesbian Internalized Homophobia Scale. Psychology in Russia: State of the Art, 10(2), 5–20. https://doi.org/10.11621/pir.2017.0201

Nguyen, T. Q., Poteat, T., Bandeen-Roche, K., German, D., Nguyen, Y. H., Vu, L. K.‑C., Nguyen, N. T.‑T., & Knowlton, A. R. (2016). The Internalized Homophobia Scale for Vietnamese Sexual Minority Women: Conceptualization, Factor Structure, Reliability, and Associations With Hypothesized Correlates. Archives of Sexual Behavior, 45(6), 1329–1346. https://doi.org/10.1007/s10508-016-0694-6

Pala, A. N., Dell'Amore, F., Steca, P., Clinton, L., Sandfort, T., & Rael, C. (2017). Validation of the Minority Stress Scale Among Italian Gay and Bisexual Men. Psychology of Sexual Orientation and Gender Diversity, 4(4), 451–459. https://doi.org/10.1037/sgd0000243

Torres, T. S., Luz, P. M., Bezerra, D. R. B., Almeida-Brasil, C. C., Marins, L. M. S., Veloso, V. G., Grinsztejn, B., Harel, D., & Thombs, B. D. (2021). Translation and validation in Brazilian Portuguese of the reactions to homosexuality scale. Psychology, Health & Medicine, 26(sup1), 20–36. https://doi.org/10.1080/13548506.2021.1936580

Yanykin, A. A., & Nasledov, A. D. (2017). Internalized homophobia in Russia. Psychology in Russia: State of the Art, 10(2), 103–116. https://doi.org/10.11621/pir.2017.0207

## E2E3

Augustinavicius, J. L., Baral, S. D., Murray, S. M., Jackman, K., Xue, Q.‑L., Sanchez, T. H., Nowak, R. G., Crowell, T. A., Zlotorzynska, M., Olawore, O., Lyons, C. E., Njindam, I. M [Iliassou M.], Tamoufe, U., Diouf, D., Drame, F., Kouanda, S., Kouame, A., Charurat, M. E., Anato, S., . . . Kane, J. C. (2020). Characterizing Cross-Culturally Relevant Metrics of Stigma Among Men Who Have Sex With Men Across 8 Sub-Saharan African Countries and the United States. American Journal of Epidemiology, 189(7), 690–697. https://doi.org/10.1093/aje/kwz270

Barros, A. C. de, & Morrison, T. G. (2023). Development and validation of the Positive Bisexual Identity (PBI) scale. Psychology & Sexuality, 14(1), 73–93. https://doi.org/10.1080/19419899.2022.2057868

Braitman, A. L., Romano, K. A., Heron, K. E., Ehlke, S. J., Shappie, A. T., & Lewis, R. J. (2021). Psychometric analysis and validity of the Daily Sexual Minority Stressors Scale among young adult same-sex female couples. Psychological Assessment, 33(11), 1025–1037. https://doi.org/10.1037/pas0001073

Brewster, M. E., & Moradi, B. (2010). Perceived experiences of anti-bisexual prejudice: Instrument development and evaluation. Journal of Counseling Psychology, 57(4), 451–468. https://doi.org/10.1037/a0021116

Burns, M. N., Kamen, C., Lehman, K. A., & Beach, S. R. H. (2012). Minority Stress and Attributions for Discriminatory Events Predict Social Anxiety in Gay Men. Cognitive Therapy and Research, 36(1), 25–35. https://doi.org/10.1007/s10608-010-9302-6

Del Pino, H. E., Steers, W. N., Lee, M., McCuller, J., Hays, R. D., & Harawa, N. T. (2022). Measuring Gender Role Conflict, Internalized Stigma, and Racial and Sexual Identity in Behaviorally Bisexual Black Men. Archives of Sexual Behavior, 51(2), 1019–1030. https://doi.org/10.1007/s10508-021-01925-w

Dyar, C., Feinstein, B. A., Eaton, N. R., & London, B. (2016). Development and Initial Validation of the Sexual Minority Women Rejection Sensitivity Scale. Psychology of Women Quarterly, 40(1), 120–137. https://doi.org/10.1177/0361684315608843

Dyar, C., Feinstein, B. A., & Davila, J. (2019). Development and Validation of a Brief Version of the Anti-Bisexual Experiences Scale. Archives of Sexual Behavior, 48(1), 175–189. https://doi.org/10.1007/s10508-018-1157-z

Flanders, C. E., LeBreton, M., & Robinson, M. (2019). Bisexual Women's Experience of Microaggressions and Microaffirmations: A Community-Based, Mixed-Methods Scale Development Project. Archives of Sexual Behavior, 48(1), 143–158. https://doi.org/10.1007/s10508-017-1135-x

Fredrick, E. G. Development and Validation of the Bisexual Microaggressions Scale. East T ennessee State University. https://dc.etsu.edu/cgi/viewcontent.cgi?article=4735&context=etd

Heron, K. E., Braitman, A. L., Lewis, R. J., Shappie, A. T., & Hitson, P. T. (2018). Measuring Sexual Minority Stressors in Lesbian Women's Daily Lives: Initial Scale Development. Psychology of Sexual Orientation and Gender Diversity, 5(3), 387–395. https://doi.org/10.1037/sgd0000287

Korhonen, C. J., Flaherty, B. P., Wahome, E., Macharia, P., Musyoki, H., Battacharjee, P., Kimani, J., Doshi, M., Mathenge, J., Lorway, R. R., Sanders, E. J., & Graham, S. M. (2022). Validity and reliability of the Neilands sexual stigma scale among Kenyan gay, bisexual, and other men who have sex with men. BMC Public Health, 22(1), 754. https://doi.org/10.1186/s12889-022-13066-3

Logie, C. H., & Earnshaw, V. (2015). Adapting and validating a scale to measure sexual stigma among lesbian, bisexual and queer women. PloS One, 10(2), e0116198. https://doi.org/10.1371/journal.pone.0116198

Maiolatesi, A. J., Satyanarayana, S., Bränström, R., & Pachankis, J. E. (2023). Development and Validation of Two Abbreviated Intraminority Gay Community Stress Scales. Assessment, 30(1), 84–101. https://doi.org/10.1177/10731911211042933

Maiolatesi, A. J., Wang, K., Burton, C. L., Harkness, A., Esserman, D. A., Safren, S. A., & Pachankis, J. E. (2023). Rejection Sensitivity and Sexual Minority Men's Social Anxiety Disorder: The Moderating Role of Sexual Identity Strength. Self and Identity : The Journal of the International Society for Self and Identity, 22(4), 563–591. https://doi.org/10.1080/15298868.2022.2125062

Murray, S. M., Wiginton, J. M., Xue, Q. L., Dibble, K., Sanchez, T., Kane, J. C., Augustinavicius, J., Nowak, R. G., Crowell, T. A., Njindam, I. M [Iliassou Mfochive], Tamoufe, U., Charurat, M., Turpin, G., Sithole, B., Mothopeng, T., Nemande, S., Simplice, A., Kouanda, S., Diouf, D., . . . Baral, S. (2024). Measuring sexual behavior stigma among cisgender men who have sex with men: An assessment of cross-country measurement invariance. Stigma and Health, 9(3), 349–361. https://doi.org/10.1037/sah0000443

Pachankis, J. E., Clark, K. A., Burton, C. L., Hughto, J. M. W., Bränström, R., & Keene, D. E. (2020). Sex, status, competition, and exclusion: Intraminority stress from within the gay community and gay and bisexual men's mental health. Journal of Personality and Social Psychology, 119(3), 713–740. https://doi.org/10.1037/pspp0000282

Pachankis, J. E., Goldfried, M. R., & Ramrattan, M. E. (2008). Extension of the rejection sensitivity construct to the interpersonal functioning of gay men. Journal of Consulting and Clinical Psychology, 76(2), 306–317. https://doi.org/10.1037/0022-006X.76.2.306

Paul, R., Smith, N. G., Mohr, J. J., & Ross, L. E. (2014). Measuring dimensions of bisexual identity: Initial development of the Bisexual Identity Inventory. Psychology of Sexual Orientation and Gender Diversity, 1(4), 452–460. https://doi.org/10.1037/sgd0000069

Rodney Lemery. (2006). Development and Validation of the Internalized Sexual Prejudice Scale. https://www.researchgate.net/profile/Rodney-Lemery/publication/267749023_Development_and_Validation_of_the_Internalized_Sexual_Prejudice_Scale/links/5458fc750cf2cf516483c651/Development-and-Validation-of-the-Internalized-Sexual-Prejudice-Scale.pdf https://doi.org/10.13140/2.1.3176.2882

Ross, M. W [Michael W.], Smolenski, D. J., Kajubi, P., Mandel, J. S., McFarland, W., & Raymond, F. H. (2010). Measurement of internalized homonegativity in gay and bisexual men in Uganda: Cross-cultural properties of the Internalized Homonegativity scale. Psychology, Health & Medicine, 15(2), 159–165. https://doi.org/10.1080/13548500903527746

Shepherd, B. F., Maki, J. L., Zelaya, D. G., Warner, Ş., Wilson, A., & Brochu, P. M. (2023). Development and Validation of the Gay-Specific Intraminority Stigma Inventory (G-SISI): Initial Evidence Underpinned by Intraminority Stress Theory. European Journal of Investigation in Health, Psychology and Education, 13(1), 170–186. https://doi.org/10.3390/ejihpe13010013

Socall, D. W. Shame, pride, and substance abuse proclivity in gay men: A theory-based investigation. https://d-legacy.lib.msu.edu/etd/29877/datastream/OBJ/view

van Dam, M. A. (2015). Alida-II: An Improved Disclosure Scale. Journal of Homosexuality, 62(10), 1313–1328. https://doi.org/10.1080/00918369.2015.1060052

Zarwell, M., Walsh, J. L., Quinn, K. G., Kaniuka, A., Patton, A., Robinson, W. T., & Cramer, R. J. (2021). A psychometric assessment of a network social capital scale among sexual minority men and gender minority individuals. BMC Public Health, 21(1), 1918. https://doi.org/10.1186/s12889-021-11970-8

## E2E4

Balsam, K. F., Molina, Y., Beadnell, B., Simoni, J., & Walters, K. (2011). Measuring multiple minority stress: The LGBT People of Color Microaggressions Scale. Cultural Diversity & Ethnic Minority Psychology, 17(2), 163–174. https://doi.org/10.1037/a0023244

Goldbach, C., & Knutson, D. (2023). Gender-related minority stress and gender dysphoria: Development and initial validation of the Gender Dysphoria Triggers Scale (GDTS). Psychology of Sexual Orientation and Gender Diversity, 10(3), 383–396. https://doi.org/10.1037/sgd0000548

Heron, K. E., Braitman, A. L., Lewis, R. J., Shappie, A. T., & Hitson, P. T. (2018). Measuring Sexual Minority Stressors in Lesbian Women's Daily Lives: Initial Scale Development. Psychology of Sexual Orientation and Gender Diversity, 5(3), 387–395. https://doi.org/10.1037/sgd0000287

Huynh, K. D., Bricker, N. L., Lee, D. L., & Balsam, K. F. (2022). Development and validation of the LGBTQ+ POC Microaggressions Scale-Brief (LGBTQ+ PCMS-B). Stigma and Health, Advance online publication. https://doi.org/10.1037/sah0000419

Liddle, B. J., Luzzo, D. A., Hauenstein, A. L., & Schuck, K. (2004). Construction and Validation of the Lesbian, Gay, Bisexual, and Transgendered Climate Inventory. Journal of Career Assessment, 12(1), 33–50. https://doi.org/10.1177/1069072703257722

Lin, Y., & Israel, T [Tania] (2012). DEVELOPMENT AND VALIDATION OF A PSYCHOLOGICAL SENSE OF LGBT COMMUNITY SCALE. Journal of Community Psychology, 40(5), 573–587. https://doi.org/10.1002/jcop.21483

Resnick, C. A., & Galupo, M. P. (2016). Assessing Experiences With LGBT Microaggressions in the Workplace: Development and Validation of the Microaggression Experiences at Work Scale. ProQuest LLC.

Woodford, M. R., Chonody, J. M., Kulick, A., Brennan, D. J., & Renn, K. (2015). The LGBQ Microaggressions on Campus Scale: A Scale Development and Validation Study. Journal of Homosexuality, 62(12), 1660–1687. https://doi.org/10.1080/00918369.2015.1078205

Zelaya, D. G., DeBlaere, C., & Velez, B. L. (2023). Psychometric validation and extension of the LGBT people of Color Microaggressions Scale with a sample of sexual minority BIPOC college students. Psychology of Sexual Orientation and Gender Diversity, 10(3), 405–412. https://doi.org/10.1037/sgd0000553

## E3E4

Bruce, D., Harper, G. W., & Bauermeister, J. A. (2015). Minority STRESS, POSITIVE IDENTITY DEVELOPMENT, AND DEPRESSIVE SYMPTOMS: Implications FOR RESILIENCE AMONG SEXUAL MINORITY MALE YOUTH. Psychology of Sexual Orientation and Gender Diversity, 2(3), 287–296. https://doi.org/10.1037/sgd0000128

## E1E2E3

Ha, H., Ross, M. W [Michael W.], Risser, J. M. H., & Nguyen, H. T. M. (2013). Measurement of Stigma in Men Who Have Sex with Men in Hanoi, Vietnam: Assessment of a Homosexuality-Related Stigma Scale. Journal of Sexually Transmitted Diseases, 2013, 174506. https://doi.org/10.1155/2013/174506

Lin, C.‑Y., Huang, Y.‑T., Lee, C.‑H., Fan, C.‑W., & Yen, C.‑F. (2022). Gay Community Stress Scale with Its Cultural Translation and Adaptions in Taiwan. International Journal of Environmental Research and Public Health, 19(18). https://doi.org/10.3390/ijerph191811649

Neilands, T. B., Steward, W. T., & Choi, K.‑H. (2008). Assessment of stigma towards homosexuality in China: A study of men who have sex with men. Archives of Sexual Behavior, 37(5), 838–844. https://doi.org/10.1007/s10508-007-9305-x

Ren, Z., & Hood, R. W. (2018). Internalized Homophobia Scale for Gay Chinese Men: Conceptualization, Factor Structure, Reliability, and Associations With Hypothesized Correlates. American Journal of Men's Health, 12(5), 1297–1306. https://doi.org/10.1177/1557988318768603

Wei, C., & Israel, T [T.] (2023). The Chinese Internalized Binegativity Scale: Measure development and cultural adaptation. Journal of Counseling Psychology, 70(6), 657–670. https://doi.org/10.1037/cou0000707

Wiginton, J. M., Murray, S. M., Algarin, A. B., Baral, S. D., Sanchez, T. H., & Smith, L. R. (2022). Metrics of sexual behavior stigma among cisgender men who have sex with men in Mexico: Exploratory and confirmatory factor analyses. BMC Infectious Diseases, 22(1), 690. https://doi.org/10.1186/s12879-022-07672-0

## E1E2E4

Rivero-Díaz, M. L., Agulló-Tomás, E., & Llosa, J. A. (2021). Adaptation and Validation of the LGBTCI to the Spanish LGBT Working Population. Journal of Career Assessment, 29(3), 422–441. https://doi.org/10.1177/1069072720982339

## E1E3E4

Delgado, J. B., & Castro, M. C. (2014). Construction and Validation of a Subjective Scale of Stigma and Discrimination (SISD) for the Gay Men and Transgender Women Population in Chile. Sexuality Research and Social Policy, 11(3), 187–198. https://doi.org/10.1007/s13178-014-0150-0

## E2E3E4

Frost, D. M., & Meyer, I. H. (2023). Minority stress theory: Application, critique, and continued relevance. Current Opinion in Psychology, 51, 101579. https://doi.org/10.1016/j.copsyc.2023.101579

Huang, M.‑F., Chang, Y.‑P., Lin, C.‑Y., & Yen, C.‑F. (2022). A Newly Developed Scale for Assessing Experienced and Anticipated Sexual Stigma in Health-Care Services for Gay and Bisexual Men. International Journal of Environmental Research and Public Health, 19(21). https://doi.org/10.3390/ijerph192113877

Kalichman, S. C., Shkembi, B., & El-Krab, R. (2023). Geometric approach to measuring intersectional stigma among black sexual minority men: Reliability and validity in an HIV prevention context. Sexual Health, 20(5), 441–452. https://doi.org/10.1071/SH23021

Lin, C.‑Y., Chang, Y.‑P., Chou, W.‑J., & Yen, C.‑F. (2023). Assessing Enacted Sexual Stigma toward Gay and Bisexual Men in the Military: The Enacted Sexual Stigma Experiences Scale in Military Service. International Journal of Environmental Research and Public Health, 20(2). https://doi.org/10.3390/ijerph20021260

Meanley, S., Brennan-Ing, M., Cook, J. A., Brown, A. L., Haberlen, S. A., Palella, F. J., Shoptaw, S. J., Ware, D., Egan, J. E., Friedman, M. R., & Plankey, M. W. (2024). Psychometric Assessment of a Homophobia Management Scale Among Cisgender Sexual Minority Men in Midlife and Older Adulthood. Psychology of Sexual Orientation and Gender Diversity, 11(2), 316–327. https://doi.org/10.1037/sgd0000600

Paul, Z. A. (2023). Bisexual+ women of color and microaffirmations.Dp - 2023. Dissertation Abstracts International: Section B: The Sciences and Engineering, 84(1-B), No Pagination Specified. https://doi.org/Study

Puckett, J. A., Surace, F. I., Levitt, H. M., & Horne, S. G [S. G.] (2016). Sexual Orientation Identity in Relation to Minority Stress and Mental Health in Sexual Minority Women. Lgbt Health, 3(5), 350–356. https://doi.org/10.1089/lgbt.2015.0088

## E1E2E3E4

Gilbert, P. A., & Rhodes, S. D. (2012). Psychometric Performance of a Novel Measure of Social Support Among Spanish-Speaking Immigrant Latino Gay Men. Hispanic Journal of Behavioral Sciences, 34(3), 491–504. https://doi.org/10.1177/0739986312446290

Liu, H., Feng, T., & Rhodes, A. G. (2009). Assessment of the Chinese version of HIV and homosexuality related stigma scales. Sexually Transmitted Infections, 85(1), 65–69. https://doi.org/10.1136/sti.2008.032714
